# Continuous chest compressions are associated with higher peak inspiratory pressures when compared to 30:2 in an experimental cardiac arrest model

**DOI:** 10.1186/s40635-023-00559-7

**Published:** 2023-11-08

**Authors:** Johan Mälberg, Silvia Marchesi, Douglas Spangler, Nermin Hadziosmanovic, David Smekal, Sten Rubertsson

**Affiliations:** 1 Department of Surgical Sciences-Anesthesia and Intensive Care, Uppsala University, Uppsala, Sweden; 2 Division of Intensive- and Perioperative Care, Skåne University Hospital, Malmö, Sweden; 3 Uppsala Clinical Research Center, Uppsala University, Uppsala, Sweden

**Keywords:** Cardiac arrest, Cardiopulmonary resuscitation, Ventilation, Animal model, Lung injuries

## Abstract

**Background:**

Ventilation during cardiopulmonary resuscitation (CPR) has long been a part of the standard treatment during cardiac arrests. Ventilation is usually given either during continuous chest compressions (CCC) or during a short pause after every 30 chest compressions (30:2). There is limited knowledge of how ventilation is delivered if it effects the hemodynamics and if it plays a role in the occurrence of lung injuries. The aim of this study was to compare ventilation parameters, hemodynamics, blood gases and lung injuries during experimental CPR given with CCC and 30:2 in a porcine model.

**Methods:**

Sixteen pigs weighing approximately 33 kg were randomized to either receive CPR with CCC or 30:2. Ventricular fibrillation was induced by passing an electrical current through the heart. CPR was started after 3 min and given for 20 min. Chest compressions were provided mechanically with a chest compression device and ventilations were delivered manually with a self-inflating bag and 12 l/min of oxygen. During the experiment, ventilation parameters and hemodynamics were sampled continuously, and arterial blood gases were taken every five minutes. After euthanasia and cessation of CPR, the lungs and heart were removed in block and visually examined followed by sampling of lung tissue which were examined using microscopy.

**Results:**

In the CCC group and the 30:2 group, peak inspiratory pressure (PIP) was 58.6 and 35.1 cmH_2_O (*p* < 0.001), minute volume (MV) 2189.6 and 1267.1 ml (*p* < 0.001), peak expired carbon dioxide (PECO_2_) 28.6 and 39.4 mmHg (*p* = 0.020), partial pressure of carbon dioxide (PaCO_2_) 50.2 and 61.1 mmHg (*p* = 0.013) and pH 7.3 and 7.2 (*p* = 0.029), respectively. Central venous pressure (CVP) decreased more over time in the 30:2 group (*p* = 0.023). All lungs were injured, but there were no differences between the groups.

**Conclusions:**

Ventilation during CCC resulted in a higher PIP, MV and pH and lower PECO_2_ and PaCO_2_, showing that ventilation mode during CPR can affect ventilation parameters and blood gases.

**Supplementary Information:**

The online version contains supplementary material available at 10.1186/s40635-023-00559-7.

## Introduction

Ventilation during cardiopulmonary resuscitation (CPR) is considered vital and should be performed as soon as possible by providers of advanced life support according to current guidelines [1]. Despite ventilation having been a part of CPR from the early days of the intervention [2], the evidence supporting the guidelines is limited and the optimal rate, volume, and airway pressure remains largely unknown [3].

Per current guidelines, the inspiratory volume should be given over 1 s and produce a visible chest rise [1]. This corresponds to on average 380 ml in adults [4]. The rate of ventilations depends on the mode of CPR used. If a bag-valve-mask is used, 30 compressions are followed by a pause during which 2 ventilations are given (30:2). This corresponds to approximately 6 breaths/minute. If an advanced airway is used, such as a supraglottic airway (SGA) or endotracheal tube (ET), ventilations can be given during continuous chest compressions (CCC) at a rate of 10 breaths/min, largely based on expert opinion [1]. There are no recommendations for optimal airway pressure in the current guidelines.

There is substantial heterogeneity in the clinical practice of ventilation according to survey studies. Many respondents reported often disregarding guidelines when ventilating. Different modes were reported, among them ventilation during CCC, 30:2, mechanical ventilation, and no ventilation at all [5, 6]. In other clinical and experimental studies, hyperventilation has been found to be common [7, 8]. This could be detrimental to the patients circulation, as high ventilation frequency and large tidal volumes have been linked to high intrathoracic pressure with lower venous return and coronary perfusion pressure [3, 9].

When comparing 30:2 and CCC in out-of-hospital cardiac arrest patients, one large study found no difference in the survival rates between the groups. There were, however, some differences in secondary outcomes in favor of 30:2, such as a higher rate of survival to hospital admission and more hospital free survival days [10]. These findings suggest that the ventilation mode might impact patient outcomes.

The physiological and pathological aspects of ventilation during CPR are not fully understood [3]. In patients hospitalized for reasons other than cardiac arrest, such as acute respiratory distress syndrome (ARDS), it has been shown that high tidal volumes and airway pressures increase the likelihood of lung injuries such as barotrauma and mortality [11, 12]. A peak inspiratory pressure of 30 cmH_2_O is commonly used as a protective threshold in ventilated patients. Interestingly, a pilot study measuring ventilation during OHCA found airway pressures to be generally higher when ventilations were given during chest compressions compared to when given during a pause in compressions. The same study also found that the inspiratory pressure reached in CCC mode often exceeded the safe threshold of 30 cm H_2_O. Tidal volumes delivered during ongoing chest compressions were also generally lower than volumes delivered during a pause [13].

Clinical studies on ventilation during cardiac arrest often comprise a large variability in treatment and patient population leading to a limited ability to identify the physiological mechanisms behind the treatment effects of different ventilation methods. Experimental studies have a major role to play in eliminating confounding factors and variability, providing a detailed picture of physiological impacts, and focusing on ventilation as the intervention of interest.

The aim of this experimental trial was to investigate the differences in lung ventilation, hemodynamics, and lung injuries between CCC and 30:2 CPR modes in a porcine model.

## Methods

This randomized experimental trial was conducted at the Hedenstierna Laboratory (Uppsala University, Sweden) and is reported in accordance with the Arrive 2.0 Guidelines [14] and a checklist is included as Additional file 1. The study was approved by the Animal Ethics Board in Uppsala, Sweden (Dnr. 5.8.18-05377/2021).

Healthy pigs aged 3–5 months of the Norwegian landrace/Yorkshire/Hampshire mixed breed, were included in the experiment. The pigs were randomized into two groups at a 1:1 ratio using the Research Randomizer online software [15] prior to the start of the experiments. One group of pigs received continuous chest compressions and continuous ventilations (10/min) asynchronous with the chest compressions (CCC group). In this group, a timer was used to give a signal every 6th second to indicate when the ventilations were to be given (corresponding to 10 ventilations/min). The second group received 30 chest compressions followed by a 3.2 s pause, during which 2 ventilations were given (corresponding to 6 ventilations/min) (30:2 group). The chest compressions in both groups were given with a frequency of 102/min using a LUCAS 3 mechanical chest compression device (Jolife AB/Stryker, Lund, Sweden).

A Laerdal Bag II (maximum measured volume: 1650 ml) (Laerdal medical, Stavanger, Norway) was used for the manual ventilation. The same person ventilated all of the pigs in the study. A portable respiratory monitor based on pneumotachography (Fluxmed GrH®, MBMed, Buenos Aires, Argentina) was used to both record the ventilation data and guide the ventilations with the aim of each ventilation being as close to the intended tidal volume of 8 ml/kg of weight as possible.

Due to the nature of this study, the researchers who performed the experiment were not blinded to the randomization.

### Protocol

#### Preparation

The pigs fasted for 12 h before the experiment, with free access to water. Upon the arrival to the laboratory, they were weighed, and their status checked (signs of stress, wounds or dehydration). They were then anesthetized with a subcutaneous injection of tiletamine (6 mg/kg) and zolazepam (2.2 mg/kg). After 5 to 10 min, they were placed on an operating table where an ear vein was cannulated to administer a bolus of fentanyl (10–20 mg/kg). The pigs were tracheotomized and an endotracheal tube inserted. One neck artery and one femoral artery were cannulated (one was connected to the monitor through a transducer for pressure measurements and the other was used to withdraw blood gas samples). A jugular vein was also cannulated and connected to the monitor to facilitate central venous pressure measurements. A small incision was performed to expose the urinary bladder for insertion of a urinary catheter.

Anesthesia was maintained throughout the experiment with a continuous infusion of ketamine (30 mg kg^−1^ h^−1^), midazolam (0.1 mg kg^−1^ h^−1^) and fentanyl (3.75 mg kg^−1^ h^−1^). After checking that the anesthesia depth was sufficient to prevent responses to painful stimuli, a continuous infusion of rocuronium (following a bolus dose of 0.6 mg/kg) was started. During the one-hour preparation time an infusion of Ringer’s acetate was administered with 30 ml kg^−1^ h^−1^ to prevent dehydration. Mechanical ventilation was performed (Servo I, Maquet, Stockholm, Sweden) in volume-controlled mode with Vt 6-8 ml/kg, respiratory rate 25, inspiratory:expiratory ratio 1:2, FiO_2_ 0.5 and PEEP 5 cmH_2_0. At the end of the surgical preparation, the pigs were left to rest for 30 min.

#### Experiment

Before the induction of cardiac arrest, the mechanical chest compression device was placed on the pigs’ thorax. To ensure adhesion of the suction cup, the edge of the cup was glued to the chest using handball glue. Once the device was positioned, the pig’s hooves were fixated to the table using tape and sandbags were placed between the pig’s flanks and the legs of the device, to prevent any unwanted movement. A baseline arterial blood gas (ABG) was taken.

Ventricular fibrillation (VF) was induced by passing an alternating current of 200 mA for 5 s through the heart of the pig. VF was verified on the electrocardiogram and by the loss of arterial blood pressure.

The pigs were then left untreated for 3 min. During these 3 min the ventilator was disconnected, the portable respiratory monitor and ventilation bag were connected to the endotracheal tube and oxygen connected to the bag with a flow of 12 l/min.

**Fig. 1:**
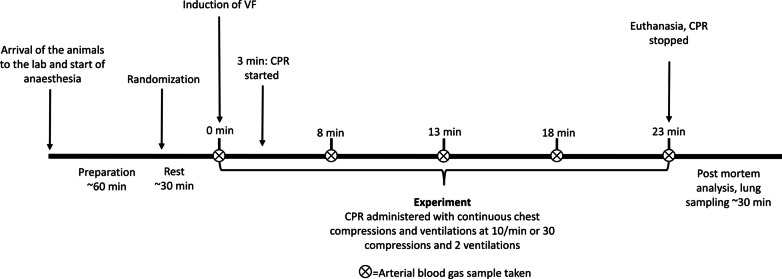
Timeline of the protocol. *CPR* cardiopulmonary resuscitation, *VF* ventricular fibrillation

CPR was started according to the randomization. Every 5 min, an ABG sample was taken. Arterial pressure, central venous pressure and ventilation parameters were monitored and recorded continuously during the experiment. After 20 min of CPR, the pigs were euthanized with an injection of potassium chloride (KCl). The chest compressions were continued for a short time after the administration of KCl, to facilitate proper perfusion of the heart. The timeline of the protocol can be found in Fig. 1.

### Data curation

#### Ventilation data

The portable respiratory monitor was used for monitoring and recording of ventilation data at a rate of 256 Hz. The device measured time, gas flow, respiratory volume, airway pressure, and exhaled CO_2_. An R-script (Additional file 2) was written to extract the peak values for each of these parameters as well as the duration of the inspiration for each individual ventilation resulting in the following parameters: peak inspiratory flow (PIF), inspiratory tidal volume (Vti), peak inspiratory pressure (PIP), duration of inspiration (Ti) and peak expiratory CO_2_ (PECO_2_). PECO_2_ was used as a proxy for EtCO_2_.

A mean value was then calculated for each ventilation parameter/minute of the experiment (20 in total/parameter) and used for the analysis.

SpO_2_ was monitored before the induction of cardiac arrest and was included in the baseline values.

#### Hemodynamic data

Arterial pressure and central venous pressure were monitored continuously throughout the experiment and recorded at 125 Hz.

Data from 5 min before the induction of cardiac arrest were used to calculate Baseline values. Heart rate was included only as a baseline measurement as the chest compression device performed chest compressions with a standard frequency of 102 per minute throughout the experiment.

For each compression/decompression cycle, two time points were identified, maximum compression and maximum decompression. At the time of maximum compression, maximum arterial pressure (maxAP) and maximum central venous pressure (maxCVP) were recorded. At the time of maximum decompression, minimum arterial pressure (minAP) and minimum central venous pressure (minCVP) were recorded. In order to identify these values, a MatLab script was written and applied to the collected data (Additional file 3).

Coronary perfusion pressure (CPP) was calculated at each compression/decompression cycle as the difference between arterial and central venous pressure at the end of decompression [16]. A mean value per minute was then calculated for each hemodynamic parameter and used for the analysis.

### Post-mortem analysis

#### Necroscopic examination

After the experiment all animals underwent a necroscopic examination and the incidence of injuries were recorded. A more in-depth assessment of the lungs was also performed, with the visual grading of atelectasis, signs of hyperinflation and hemorrhages (graded 0 to 3). The examination and grading system to assess the injuries are described in detail in Additional file 4.

#### Histopathological analysis

After the necroscopic examination, lung tissue samples were taken from 5 parts of both the left and right lung and put in formaldehyde. The sampled locations were: paracardiac region, upper ventral lobe, upper dorsal lobe, lower ventral lobe and lower dorsal lobe (10 samples per subject). The samples were then shipped to the National Veterinary Institute (SVA—Uppsala, Sweden) where a pathologist, blinded to the protocol, examined them with microscopy and evaluated the following features:

1. Atelectasis (percentage of atelectasis tissue over the whole tissue analyzed in the sample, and atelectasis pattern—patchy or homogeneous).
2. Hyperinflation (percentage of hyperinflated tissue over the whole tissue analyzed in the sample).
3. Edema (assessed using a grading system from 0 to 5).
4. Microscopic hemorrhages (only the presence or absence of the feature was reported, as a binary variable).

A full description of the histopathological analysis can be found in Additional file 5**.**

#### Wet–dry ratio

The degree of edema in the lung tissue was also measured using wet–dry ratio. Samples were taken from the same locations as for the histopathological examination. They were weighed fresh, dried in an oven at 37° for a week, and then re-weighed.

### Study variables

- Peak inspiratory pressure (PIP).
- Max and min arterial pressure (maxAP, minAP).
- Max and min central venous pressure (maxCVP, minCVP).
- Coronary perfusion pressure (CPP).
- Inspiratory tidal volume (Vti).
- Duration of inspiration (Ti).
- Minute volume (MV).
- Peak expiratory CO_2_ (PECO_2_) (as a measurement of both hemodynamics and ventilation).
- Peak inspiratory flow (PIF).
- Arterial blood gases (ABG).
- Changes over time in hemodynamics, PECO_2_ and ABG during the experiment.
- Edema assessed by wet–dry ratio.
- Histopathological assessment of the lungs (atelectasis, edema and hyperinflation).

### Statistical analysis

Baseline values and the values from the post mortem analyses were based on one value per pig and analyzed using the Mann–Whitney *U*-test for continuous variables and Fishers’ exact test for categorical variables. Descriptive statistics were presented as n for categorical variables and median and IQR for continuous variables. The multiple comparisons of lung tissues were adjusted using the Bonferroni correction.

Differences in the mean values throughout the experiment and changes over time were analyzed using linear mixed models with time as a repeated measure and CPR mode, time, and CPR mode-time interaction as fixed factors. The covariance structure for the models was chosen based on analysis of model fit using Akaike’s information criterion (AIC). First order ante-dependence was found to minimize AIC in a plurality of the models and was therefore used in all the mixed models. Comparison of model fits using various covariance structures may be found in Additional file 6*.* Mean values, confidence intervals, and *p*-values were derived from the models fixed effect estimates.

Correlations were analyzed with Pearson’s test with one mean value per parameter and subject being used.

A *p*-value of < 0.05 was considered significant.

Statistical analyses performed using SPSS 28.0 (IBM©).

## Results

Eighteen pigs of both sexes were randomized to the CCC/30:2 groups. Two subjects, one from each group had major bleeding with severe impact on circulation early during the experiment and were excluded. During autopsy, ruptures in the vena cava was found in these pigs, most likely caused by the chest compressions. The remaining 16 pigs, eight in each group, were included in the study.

### Baseline

Baseline characteristics of the included subjects are described in Table 1. There were no differences between the groups.

**Table 1:** Baseline characteristics of the included subjects

	CPR mode		*p*-value^a^
	CCC (*n* = 8)	30:2 (*n* = 8)	
Female sex (*N*)	5	2	0.315
Weight (kg), median (IQR)	32.9 (29.0–35.5)	33.1 (32.0–38.0)	0.505
Vti (ml), median (IQR)^a^	278.5 (250.3–322.5)	295.5 (252.3–309.8)	0.721
PiP (cmH_2_O), median (IQR)^a^	20.5 (19.3–22.0)	19.5 (19.0–21.0)	0.442
EtCO_2_ (mmHg), median (IQR)^a^	39.8 (36.0–43.5)	43.1 (37.9–44.1)	0.382
Temperature (°C), median (IQR)^a^	38.0 (37.8–38.5)	38.3 (38.0–38.7)	0.573
HR (BPM), median (IQR)^b^	100.0 (88.0–110.5)	95.0 (75.0–104.3)	0.414
MAP (mmHg), median (IQR)^b^	91.7 (75.5–103.3)	81.8 (71.5–95.2)	0.414
CVP (mmHg), median (IQR)^b^	7.0 (1.8–9.3)	3.0 (1.0–7.0)	0.282
SpO_2_ (%), median (IQR)^a^	100.0 (99.3–100.0)	100.0 (98.5–100.0)	1.000
PaO_2_ (mmHg), median (IQR)^a^	181.9 (157.7–202.5)	183.8 (162.9–202.5)	0.798
PaCO_2_ (mmHg), median (IQR)^a^	38.0 (37.2–39.3)	40.9 (37.4–43.9)	0.279
Lactate (mmol/l), median (IQR)^a^	1.2 (1.0–1.3)	1.2 (0.8–2.3)	0.645
pH median (IQR)^a^	7.5 (7.5–7.5)	7.5 (7.4–7.5)	0.161

*IQR* interquartile range, *Vti* inspiratory tidal volume, *PIP* peak inspiratory pressure, *EtCO*_*2*_ end tidal carbon dioxide, *HR* heart rate, *MAP* mean arterial pressure,* CVP* central venous pressure,* SpO*_*2*_ peripheral oxygen saturation,* PaO*_*2*_ partial pressure of oxygen, *PaCO*_*2*_ partial pressure of carbon dioxide

^a^Last recorded value before the start of the experiment

^b^Mean value of the 5 min directly preceding the start of the experiment

### Ventilation parameters, hemodynamics and ABG

PIP was higher in the CCC group compared to the 30:2 group, 58.6 cmH_2_O, (95% CI 54.7–62.5) vs. 35.1 cmH_2_O (95% CI 31.2–38.9), 95% CI for the difference 18.0–29.0, *p* < 0.001. The remaining comparison of the mean values of ventilation parameters, hemodynamics and ABG’s for the entire experiment can be seen in Table 2

**Table 2:** Comparison of ventilation parameters, hemodynamics and ABG between the CCC- and the 30:2 group

Ventilation parameters	CPR mode		95% CI of difference between groups	*p*-value^a^
	CCC Mean (95% CI)	30:2 Mean (95% CI)		
PIF (l/min)	76.3 (72.2–80.5)	51.7 (47.6–55.9)	18.7–30.4	< 0.001*
Vti (ml)	221.5 (206.8–236.3)	221.4 (206.6–236.2)	− 20.8–21.0	0.990
Vti (ml/kg)	6.8 (6.5–7.2)	6.4 (6.1–6.8)	− 0.1–0.9	0.087
Ti (s)	0.435 (0.413–0.457)	0.419 (0.397–0.441)	(− 0.015–0.048)	0.297
MV (ml)	2189.6 (2060.8–2318.4)	1267.1 (1136.1–1398.2)	738.7–1106.2	< 0.001*
PECO_2_ (mmHg)	28.6 (22.3–34.8)	39.4 (33.1–45.6)	− 19.7–2.0	0.020*
Hemodynamics				
CPP (mmHg)	10.0 (2.5–17.4)	15.2 (7.7–22.6)	− 15.7–5.3	0.309
maxAP (mmHg)	132.4 (94.9–170.0)	108.7 (71.2–146.3)	− 29.4–76.8	0.354
minAP(mmHg)	− 4.8 (− 10.7–1.0)	− 3.2 (− 9.1–2.6)	− 9.9–6.7	0.686
maxCVP (mmHg)	169.5 (146.3–192.7)	146.4 (123.3–169.5)	− 9.6–55.9	0.153
minCVP(mmHg)	1.3 (− 5.2–7.7)	5.0 (− 1.3–11.3)	− 12.7–5.3	0.393
ABG				
PaO_2_ (mmHg)	141.7 (86.2–196.8)	156.0 (103.4–208.6)	− 90.4–61.8	0.689
PaCO_2_ (mmHg)	50.2 (44.2–56.2)	61.1 (55.5–66.7)	− 19.1–2.7	0.013*
Lactate (mmol/l)	5.6 (5.0–6.3)	5.4 (4.8–6.0)	− 0.7–1.1	0.633
pH	7.3 (7.2–7.3)	7.2 (7.2–7.3)	0.01–0.1	0.029*

*CI* confidence interval, *PIF* peak inspiratory flow, *PIP* peak inspiratory pressure, *Vti* inspiratory tidal volume, *Ti* duration of inspiration, *MV* minute volume; *p* = : peak expired carbon dioxide; *CPP* coronary perfusion pressure, *maxAP* maximum arterial pressure, *minAP* minimum arterial pressure, *maxCVP* maximum central venous pressure, *minCVP* minimum central venous pressure, *ABG* arterial blood gases, *PaO*_*2*_ partial pressure of oxygen, *PaCO*_*2*_ partial pressure of carbon dioxide

*p-value of ≤ 0.05

A positive correlation was found between PIP and CVP (*r* = 0.546, *p* = 0.029) while PIP and PECO_2_ correlated negatively (*r* = − 0.659, *p* = 0.005).

In both groups CPP, maxAP and maxCVP decreased over time (all *p* < 0.001). PaCO_2_ and Lactate increased over time while pH decreased in both groups (*p* < 0.001, *p* < 0.001 and *p* < 0.001). PaO_2_ did not change significantly during the experiment (*p* = 0.064).

**Fig. 2:**
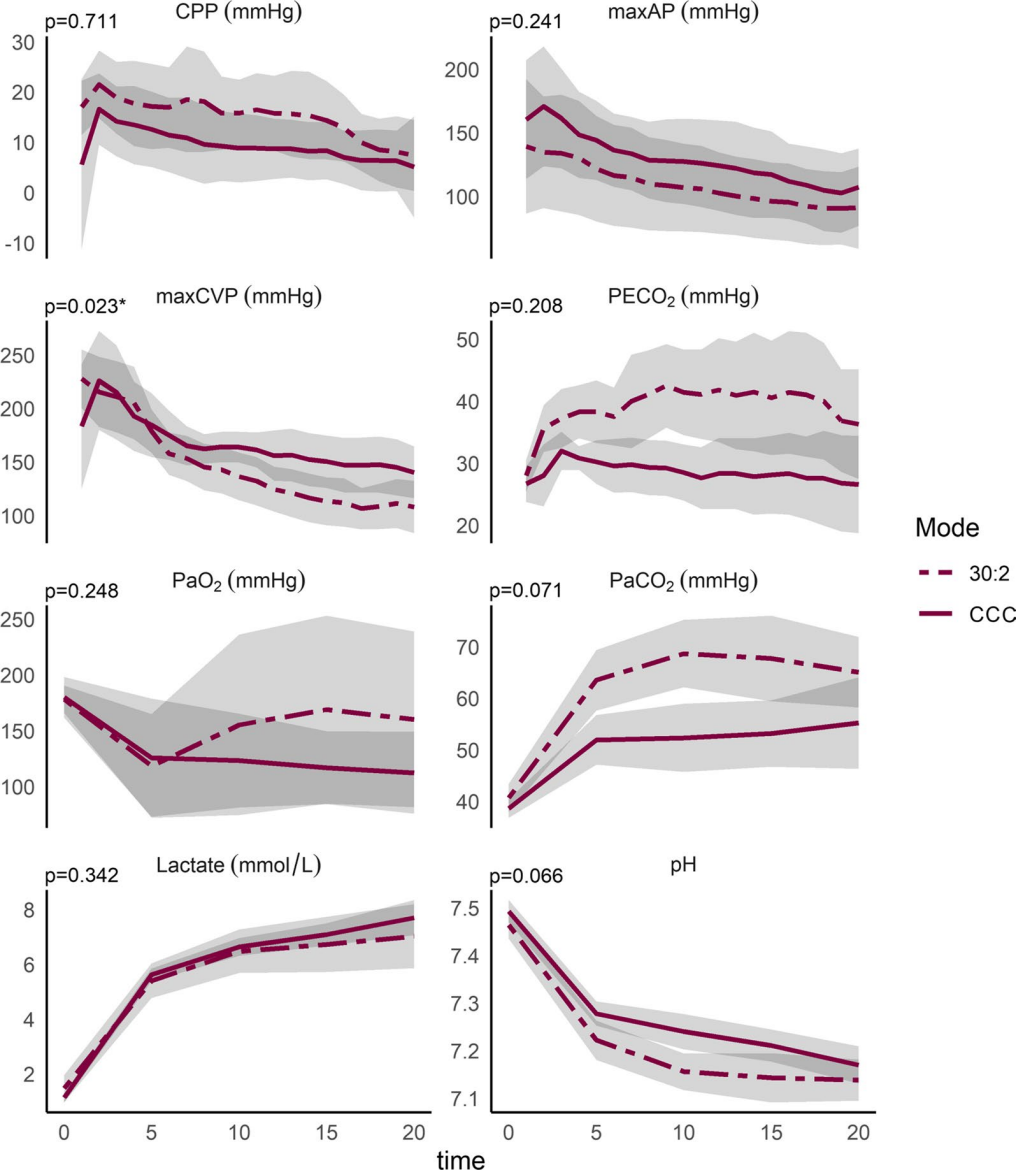
Changes over time during the experiment. The colored lines represent the mean value for each group and for each time point they were registered during the experiment. Shaded areas are 95% confidence intervals. *p*-values for the interaction term CPR-mode and time is shown. *CPP* coronary perfusion pressure, *maxAP* maximum arterial pressure, *maxCVP* maximum central venous pressure, *PECO*_*2*_ peak expired carbon dioxide, *PaO*_*2*_ partial pressure of oxygen, *PaCO*_*2*_: partial pressure of carbon dioxide. *p-value of ≤ 0.05

The changes over time can be seen in Fig. 2.

## Post-mortem analyses

**Fig. 3:**
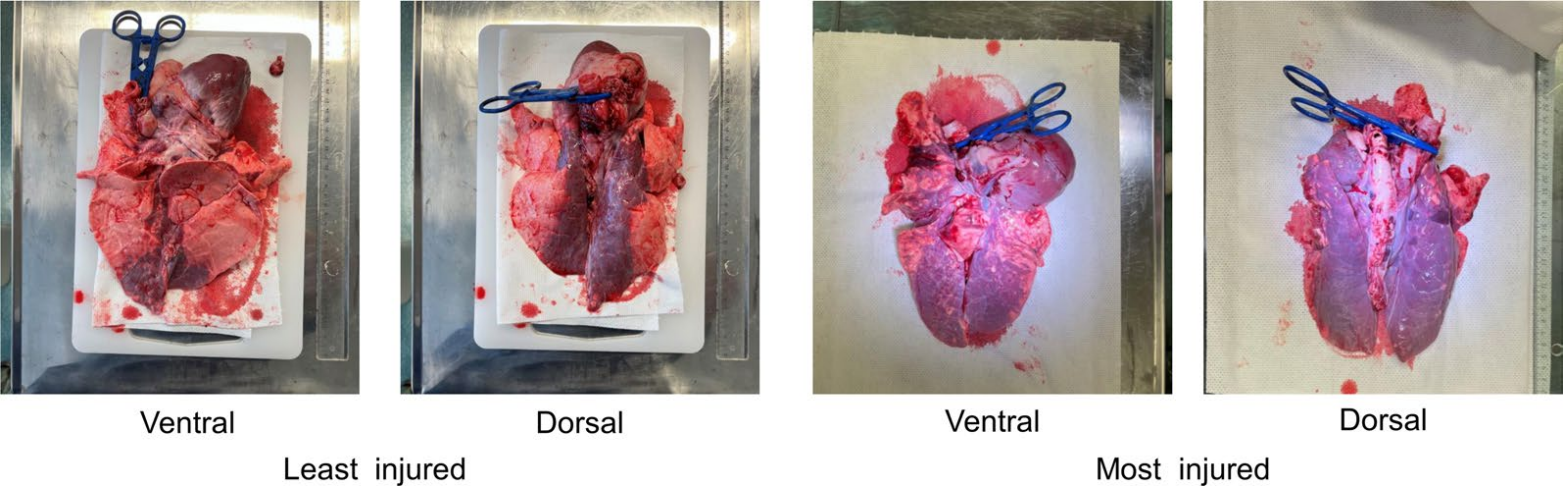
Examples of lung injuries. Least injured: CCC mode. Grading: atelectasis 1, hyperinflation 1 and macroscopic hemorrhages 0. Most injured: CCC mode. Grading: atelectasis 3, hyperinflation 3 and macroscopic hemorrhages 1

Upon visual inspection of the lungs, atelectasis was found in all of the pigs in both groups with a median grading of 2 in the CCC group and 1 in the 30:2 group. Hyperinflation was found in 8/8 in the CCC group (median grading 2) and 7/8 in the 30:2 group (median grading 1). Macroscopic hemorrhages were found in 6/8 pigs in the CCC group and 5/8 in the 30:2 group with a median grading of 1 in both groups. No significant differences were found in the incidence of lung injuries between the groups during the necroscopic examination. Examples of the least and most injured lungs can be seen in Fig. 3. All the collected lung pictures can be found in Additional file 7.

The full data and results collected during the post-mortem examination and analysis can be found in Additional file 4.

In the histopathological examination, there were no differences between the groups.

**Fig. 4:**
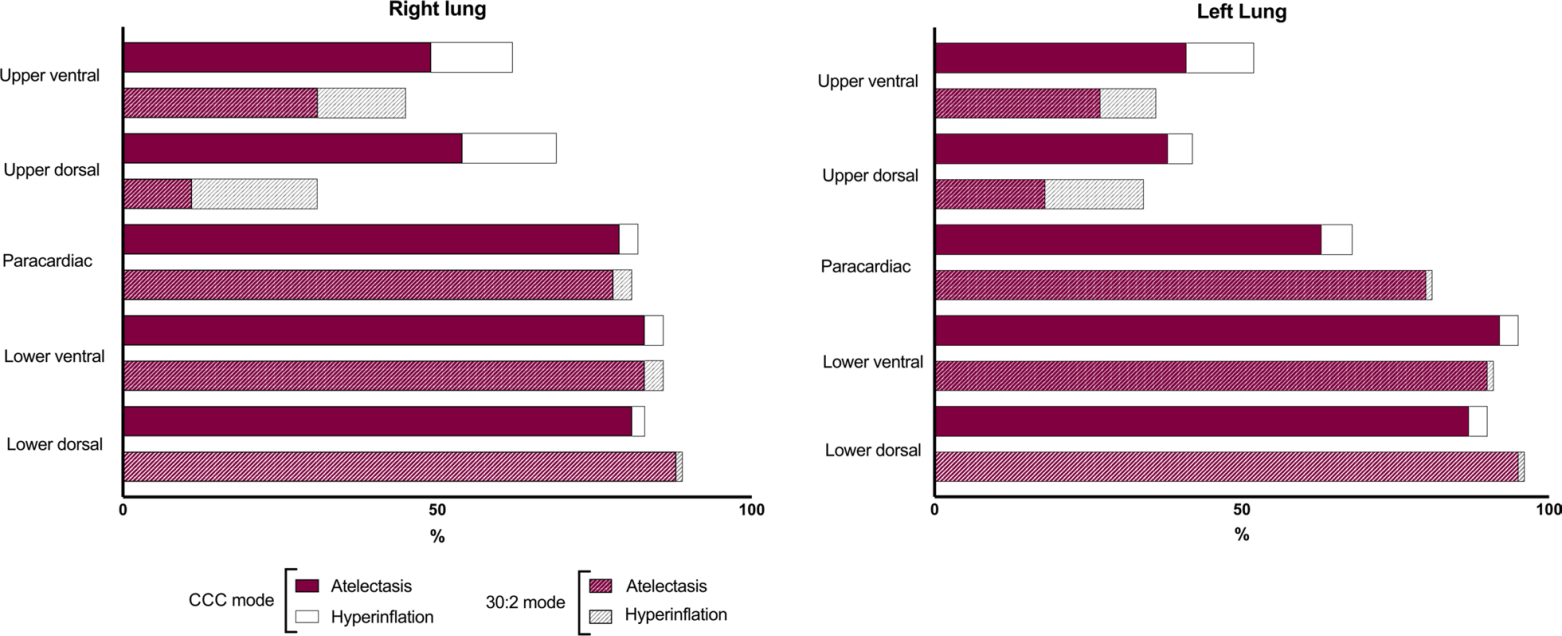
Proportion of atelectasis and hyperinflated tissue. Shown as percentage of the entire lung tissue sample

The proportion of atelectasis and hyperinflated tissue measured in the histopathological samples is shown in Fig. 4.

There were no differences in edema between the groups, either in the pathologists’ assessment or in the wet–dry ratio.

## Discussion

In this experimental study, ventilation performed during continuous chest compressions resulted in higher peak inspiratory pressures (PIP) compared to ventilation performed during pauses between compressions. One potential explanation for the higher PIP in the CCC group is that when ventilations are given asynchronous to chest compressions some of the ventilations will probably occur during the compression face of the chest compressions and thereby lead to increased intrathoracic pressure [17]. This is in line with previous findings showing a positive correlation between intrathoracic pressure and airway pressure during CPR [18]. A previous porcine experimental study with a comparable design found similar airway pressures in CCC and 30:2 mode while the tidal volumes were higher in the CCC group [19]. A possible explanation for this is larger pigs in the mentioned study, with a less compressible thorax which could have led to a lower intrathoracic pressure produced by compressions and therefore a smaller counter-pressure during ventilations. Yang et al. [13] in a clinical pilot trial found that ventilation during CCC produced a much higher airway pressure when compared to ventilation after the patient achieved ROSC (i.e., when no chest compressions were given, absolute difference 32 cmH_2_O), suggesting that in humans, airway pressure is significantly higher than normal during CPR using CCC. It should be noted though that this was not a direct comparison with ventilation during 30:2 CPR and the number of patients was limited.

In this study, a mechanical chest compression device with active decompression was used in both groups. Previous studies have shown that augmentation of a negative intrathoracic pressure can affect hemodynamics [20, 21]. However, the impact on airway pressure is less clear. One previous study found that while active decompression CPR did produce a slightly lower minimum intrapleural pressure compared to standard CPR, there were no differences in the airway pressure [18].

An inherent risk of bias exists when investigating manual ventilation. To adjust for this, a portable respiratory monitor was used to provide real-time feedback on the given ventilations, which allowed adjustments to be made continuously. The goal was to give a tidal volume as close to 8 ml/kg as possible. The results show that the ventilations were similar with regard to both Vti and Ti. Real-time feedback during ventilation has also previously been associated with better ventilation performance with tidal volumes closer to the intended goal compared to ventilations without feedback in manikins’ studies [22, 23].

The research on the impact of airway pressures and tidal volumes during CPR is limited.

An interesting aspect of the higher airway pressure in the CCC group is that in current CPR guidelines it is recommended to use CCC when an advanced airway, such as an SGA or ET is placed, unless there is considerable leakage [1]. SGA:s however, are unable to maintain a proper seal and start leaking when the airway pressure is too high. According to previous studies, this occurs when the airway pressure is between 23 and 33 cmH_2_O [24–28]. The airway pressure in the 30:2 group was just above the upper limit for this leakage threshold while the airway pressure in the CCC group greatly exceeded it. This indicates that maintaining a proper airway seal with an SGA during CCC could be difficult and the volume actually reaching the patients lungs could be affected.

The present study includes a detailed description of the lung conditions after CPR. The high incidence and large extent of atelectasis in both groups shows that CPR even during a relatively short period (20 min), can cause severe damage to healthy lungs. There are very few studies looking at post-CPR lung injuries and most of them are human cadaver studies or autopsies [29, 30]. The high incidence of post-ROSC ARDS in this study, which has also been found in other studies [31, 32] can be interpreted as confirmation that CPR has a considerable impact on the lungs. Whether ventilation mode during CPR impacts the lungs, increasing or reducing the extent of the damage, is unknown. This study found no differences in lung injuries between the groups. But as both groups had considerable injuries, a much larger sample size might be needed to identify any potential differences. Although lacking statistical significance, there were interesting trends observed in the post-mortem analysis. Among them were a larger extent of atelectasis in some areas of the lungs, higher frequency of micro-hemorrhages and more inhomogeneous atelectasis pattern in the CCC group. An inhomogeneous atelectasis pattern has been identified as a risk factor for tissue density and tissue collapse in ARDS studies [33, 34]. These trends remain speculative though and should be interpreted as merely hypothesis generating for future studies.

Another noteworthy observation is the higher PIF in the CCC group. In an experimental study on mechanically ventilated piglets, Protti et al. found that a higher PIF contributed to worse lung mechanics and more lung edema, even when the tidal volumes and plateau pressures were similar [35]. The amount of lung edema, however, did not differ between the groups in our study. Lung edema was observed by Protti et al. after 54 h of ventilation and it is possible that the present protocol was too short to show any difference in edema formation. Peak inspiratory flow (PIF) has to the authors knowledge not been studied in ventilation during CPR, but could prove to be another complicating factor when finding the most adequate ventilation mode in CPR.

PECO_2_ was significantly lower in the CCC group compared to the 30:2 group, as was PaCO_2_ while the pH was higher. This difference could be explained by the MV, which was significantly higher in the CCC group. As the subjects had similar Vti but were ventilated at a different rate depending on the group the result is a higher MV in the CCC group. Increasing the MV has long been known to decrease the PaCO_2_ in mechanically ventilated patients [36] and this interaction has also been shown with EtCO_2_ during CPR in an experimental setting [37]. Because of the dependence of ventilation, current CPR guidelines advise against using ETCO_2_ as the sole predictor of CPR outcomes [1] even though studies have shown that a low ETCO_2_, especially later during CPR, decreases the chance of survival [38, 39]. The findings in this study seem to be in line with these recommendations and perhaps suggest that ventilation mode should be taken into consideration when evaluating EtCO_2_ during CPR [40].

AP, CVP (neither max nor min) and CPP did not differ between the groups, suggesting that these parameters are not directly affected by the ventilation mode. However, a higher PIP correlated with a higher maxCVP, regardless of CPR mode. MaxCVP also decreased less over time in the CCC group compared to the 30:2 group which indicates a more persistently high maxCVP in the CCC group, especially towards the end of the experiment. The mechanism behind the correlation between high PIP and maxCVP and the less pronounced decrease of maxCVP over time in the CCC group could be due to the raised intrathoracic pressure being reflected on the vena cava pressure measurement (CVP).

### Limitations

As with all experimental animal studies, the results may not be directly applicable in humans. Pigs have an anatomy closely resembling humans with regard to the heart and lungs, although there are important differences. The pig’s thorax is more triangular in shape and the heart is more vertical compared to a human thorax and heart. This could affect the performance of chest compressions when compared to humans and its effect on hemodynamics and post-CPR injuries.

In this study, PECO_2_ was used instead of EtCO_2_. Especially in CCC mode, we found substantial noise in the CO_2_ curve caused by the chest compressions, and the exact end of each expiration could not be reliably identified. The peak value of each parameter, including the exhaled CO_2_ was therefore extracted instead. PECO_2_ is not exactly the same as EtCO_2_ and this must be taken into consideration when comparing the results of this study to previous research. Given the anomalies caused by ongoing chest compressions, PECO_2_ may be a more reliable way to find the highest CO_2_ value during expiration in this context.

As no hypothesis regarding any differences in the measured parameters and lung injuries was made beforehand, no power calculation was done and the sample size was kept low in adherence to the 3 R’s of animal studies [41]. A larger sample size could possibly have yielded clearer results.

## Conclusion

In this study, ventilation during CPR with CCC resulted in a higher PIP, MV, and pH and lower PECO_2_ and PaCO_2_ compared to ventilation with 30:2. There were no differences in lung injuries between the groups, although all lungs had considerable injuries. Further studies are needed to clarify the impact of higher airway pressure in ventilation during CCC, if it is related to the occurrence of lung injuries and if it affects outcomes in humans.

### Supplementary Information

**Additional file 1. **ARRIVE 2.0 checklist.

**Additional file 2. **R-script.

**Additional file 3. **MatLab-script.

**Additional file 4. **Post mortem examination and results.

**Additional file 5. **Histopathological analysis.

**Additional file 6. **Mixed models performance.

**Additional file 7. **Lung pictures.

## Data Availability

The datasets used and/or analyzed during the current study are available from the corresponding author on reasonable request.

## Abbreviations

ABG: Arterial blood gas
ARDS: Acute respiratory distress syndrome
AP: Arterial pressure
CCC: Continuous chest compressions
CPP: Coronary perfusion pressure
CPR: Cardiopulmonary resuscitation
CVP: Central venous pressure
ET: Endotracheal tube
EtCO_2_: End tidal carbon dioxide
HR: Heart rate
MAP: Mean arterial pressure
MV: Minute ventilation
OHCA: Out of hospital cardiac arrest
PaCO_2_: Partial pressure of carbon dioxide
PaO_2_: Partial pressure of oxygen
PECO_2_: Peak expiratory carbon dioxide
PIF: Peak inspiratory flow
PIP: Peak inspiratory pressure
ROSC: Return of spontaneous circulation
SGA: Supraglottic airway
SpO_2_: Oxygen saturation
Ti: Duration of inspiration
VF: Ventricular fibrillation
Vti: Inspiratory tidal volume
