# Supplementary material for: Continuous chest compressions are associated with higher peak inspiratory pressures when compared to 30:2 in an experimental cardiac arrest model
Source: Intensive Care Med Exp. 2023 Nov 8;11:75. doi: 10.1186/s40635-023-00559-7 (PMC10632261; doi:10.1186/s40635-023-00559-7)
Supplement: Supplementary file 3 — Additional file 3. MatLab-script. [file 40635_2023_559_MOESM3_ESM.docx]

**Matlab script for extracting maxART, maxCVP, minART and minCVP.**

maxART = maximum arterial pressure value at each compression/decompression cycle

minART = minimum arterial pressure value at each compression/decompression cycle

maxCVP = maximum central venous pressure value at each compression/decompression cycle

minCVP = minimum central venous pressure value at each compression/decompression cycle

Data were upload to Matlab as one table per subject with two columns: ART and CVP.

The first column included the arterial pressure measurements (125 values per second), and the second included the central venous pressure measurements (125 values per second).

The table was named “data” once upload.

**Script:**  **Notes**

art=data(:,1) To obtain 2 distinct column

cvp=data(:,2)

ART=art(1:150000) 150000 is the number of values registered on one case (20 min)

x=reshape(ART,73,[]) 73 is the length of a compression/decompression cycle

[MaxART, iMaxART]=max(x,[],’linear’) To obtain a 2 column table with all the maximum arterial pressure values on one column and a progressive number on the other column

Plot(MaxART(:,2), iMaxART(:,1)) To create a plot of the values

[MinART, iMinART]=min(x,[],’linear’) To obtain a 2 column table with all the minimum arterial pressure values on one column and a progressive number on the other column

Plot(MinART(:,2), iMinART(:,1)) To create a plot of the values

CVP=cvp(1:150000) 150000 is the number of values registered on one case (20 min)

y=reshape(CVP,73,[]) 73 is the length of a compression/decompression cycle

[MaxCVP, iMaxCVP]=max(y,[],’linear’) To obtain a 2 column table with all the maximum central venous pressure values on one column and a progressive number on the other column

Plot(MaxCVP(:,2), iMaxCVP(:,1)) To create a plot of the values

[MinCVP, iMinCVP]=min(y,[],’linear’) To obtain a 2 column table with all the minimum central venous pressure values on one column and a progressive number on the other column

Plot(MinCVP(:,2), iMinCVP(:,1)) To create a plot of the values

**MatLab script for calculating CPP (coronary perfusion pressure)**

CPP is calculated as arterial pressure minus central venous pressure at the end of the decompression phase.

**Script:**  **Notes**

cpp_calc=[ART-CVP] It is giving a column with 150000 values

cppindex1=iMaxART+54 The end of decompression is found 56 values after the

cppindex2=iMaxART+55 peak (maximum values of pressures), that means

cppindex3=iMaxART+56 around 0,4 sec after the end of compression. To not

cppindex4=iMaxART+57 just take 1 value, we decided to calculate a mean of

cppindex5=iMaxART+58 measurements around that precise spot.

cpp1=cpp_calc(cppindex1) We obtain 5 values for each compr/decompr cycle

cpp2=cpp_calc(cppindex2)

cpp3=cpp_calc(cppindex3)

cpp4=cpp_calc(cppindex4)

cpp5=cpp_calc(cppindex5)

cpp_data=[cpp1 cpp2 cpp3 cpp4 cpp5] We obtain a table where each row represents the cpp values calculated at the end of decompression

cppMEAN=mean(cpp_data, 2) We obtain one value of cpp per cycle (the mean of the 5 values we identified)
