# Supplementary material for: Continuous chest compressions are associated with higher peak inspiratory pressures when compared to 30:2 in an experimental cardiac arrest model
Source: Intensive Care Med Exp. 2023 Nov 8;11:75. doi: 10.1186/s40635-023-00559-7 (PMC10632261; doi:10.1186/s40635-023-00559-7)
Supplement: Supplementary file 4 — Additional file 4. Post mortem examination and results. [file 40635_2023_559_MOESM4_ESM.docx]

# Additional file 4. Post mortem examination and results

## Necroscopic examination

After the experiment a necroscopic examination was performed on all of the subjects. It was performed by two of the authors using the form shown in Table S1 as guidance.

After the potassium chloride injection, the chest compressions were stopped and asystole was confirmed by arterial line curve and ECG for around 10 minutes. The thoracic skin at the compression point was evaluated and possible marks reported. To assess possible airway bleeding (graded from 0 to 4 according to the amount of blood), suction was performed through the endotracheal tube.

The skin and subcutaneous tissues on the chest were then detached from the ribcage and sternum and eventual rib fractures were assessed and counted. The sternum was separated from the ribs from both sides and removed. Thoracic organs were observed looking for signs of big vessel lacerations, pneumothorax, hemothorax and hemoperitoneum or heart rupture. To allow for a more careful inspection of the lungs, the heart and lungs were carefully removed in-block by cutting the trachea (which had been preventively clamped), ascendant aorta, superior and inferior vena cava and pulmonary vessels. Pictures of heart and lungs were taken from a ventral and dorsal perspective (see Figure S1).

Abdomen was open with a L-shaped cut and the presence of spleen and liver laceration or other signs of hemorrhage were assessed.

**Table S1** Guidance-form to be filled in during necroscopic examination.

| **Autopsy findings** | | | | | |
| --- | --- | --- | --- | --- | --- |
| External Evaluation | | | | | |
| **Thoracic skin** | None | Light bruise | Dark bruise | Subcut/cut. haemorrhage | Scarping/abrasion |
| **Airways bleeding**  (aspiration) | None | Few stripes | Pink foam | Moderate stripes | Big amount and clots |
| **Airways bleeding**  (on the tube after extubation) | None | 1 | 2 | 3 |  |
| **Subcutaneous emphysema** | YES | | | NO | |
| Internal Evaluation | | | | | |
| **Pleural space** | Haemothorax  Dx | Haemothorax sx | Pneumothorax  Dx | Pneumothorax sx | None |
| **Sternal fracture** | None | Complete | Incomplete | Multiple | |
| **Rib fracture**  (indicate number) | Dx | | | Sx | |
| **Big vessel**  (especially vena cava) | None | | | Damage (explain) | |
| **Lungs** | None | Contusion | Laceration | Rupture |  |
| **Heart** | None | Contusion | Laceration | Rupture |  |
| **Liver** | None | Contusion | | Laceration | |
| **Spleen** | None | Contusion | | Laceration | |
| **Abdominal haemorrhage** | Yes (indicate quantity if possible) | | | No | |
| **Others** |  | | | | |

The lungs underwent a more detailed visual examination to assess three features: atelectasis, signs of hyperinflation and signs of macroscopic hemorrhages. Atelectasis were graded from 0 to 3 according to their extent (0 = no atelectasis; 1 = atelectasis visible on the caudal part of the lower lobes and on the paracardiac area; 2 = atelectasis visible on the whole lower lobes and on the paracardiac area; 3 = atelectasis that spread to the mid and upper lobes). Examples of the grading can be seen Figure S1.

Signs of hyperinflation was defined as the presence of visible emphysema bubbles on the surface of the lung.

Macroscopic hemorrhages were assessed and graded from 0 to 3 according to their extent (0 = no signs of macroscopic hemorrhages; 1 = signs of macroscopic hemorrhages with an extent that is less than 10% of the whole lung tissue; 2 = signs of macroscopic hemorrhages with an extent that is between 10 and 25% of the whole lung tissue; 3 = signs of macroscopic hemorrhages with an extent that is greater than 25% of the whole lung tissue).


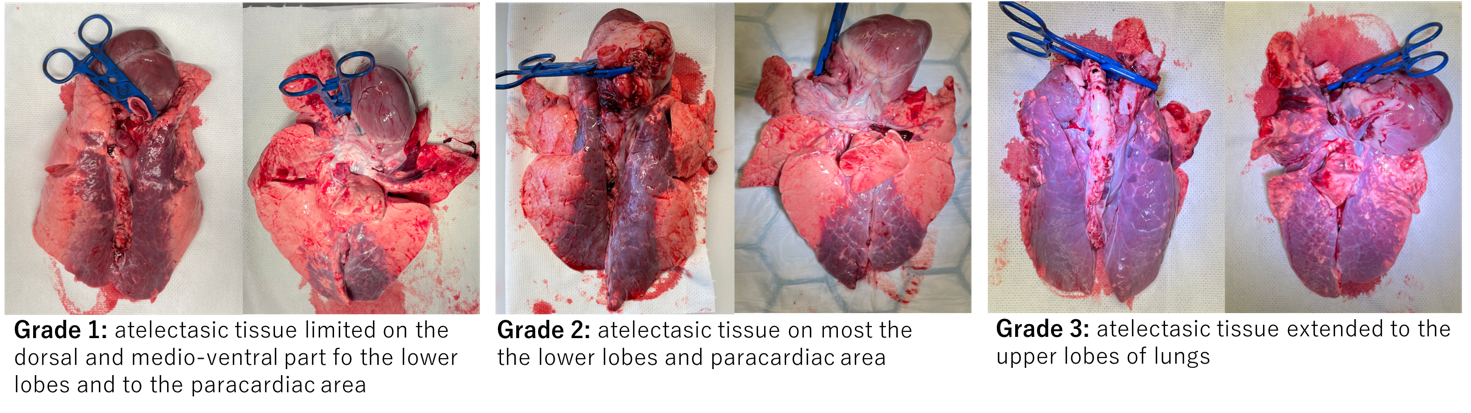
***Fig. S1*** *Atelectasis grading during necroscopic assessment.*

## Results of the necroscopic examination

There were no sternal fractures, pneumothorax, hemothorax, cardiac ruptures, thoracic vessels injuries found in any of the subject.

The results on the rest of the injuries are reported in Table S2.

**Table S2** Injuries and lung assessment from the necroscopic examination.

|  | CCC group | 30:2 group | p-value |
| --- | --- | --- | --- |
| Skin bruise | 8/8 | 8/8 | N/A |
| Airways bleeding | 4/8 | 2/8 | 0.627 |
| Airway bleeding grading – median | 1 | 1.5 | 0.573 |
| Ribs fractures | 8/8 | 8/8 | N/A |
| N. of rib fractures (right side) – median | 2 | 2.5 | 0.852 |
| N. of rib fractures (left side) - median | 3 | 2.5 | 0.573 |
| Hemopericardium | 1/8 | 1/8 | 1.000 |
| Atelectasis | 8/8 | 8/8 | N/A |
| Atelectasis grading- median | 2 | 1 | 0.234 |
| Hyperinflation | 8/8 | 7/8 | 1.000 |
| Hyperinflation grading-median | 2 | 1 | 0.234 |
| Macroscopic hemorrhages | 6/8 | 5/8 | 1.000 |
| Macroscopic hemorrhages grading- median | 1 | 1 | 0.959 |

There were no differences in the global incidence of injuries between the groups.

## Histopathological analysis

### Sampling

From each lung 5 samples, around 1.5 cm^3^, were sectioned and put in buffered formalin 4%, in 10 ml cylinders. They were extracted from 5 different anatomical regions: upper ventral lobe, upper dorsal lobe, paracardiac tissue, lower ventral lobe and lower dorsal lobe. In total 10 samples were extracted from each subject.

Once the entire experiment was completed, the samples were transferred to the National Veterinary Institute (SVA-Uppsala, Sweden) and a pathologist (blinded to the protocol) was asked to assess three features for each sample: atelectasis, indicating the percentage of the samples represented by atelectasic tissue and to describe the pattern of atelectasis (homogeneous or patchy); hyperinflation, indicating the percentage of the sample represented by hyperinflated tissue; and the presence or absence of micro-hemorrhages.

The samples were prepared by the SVA personnel on microscope slides and colored with hematoxylin-eosin. All the samples were observed as a whole at a 400X magnification.

### Results of the histopathological analysis

The percentage of atelectasis and hyperinflation are reported in the manuscript. The incidence of inhomogeneous (patchy) atelectasis pattern in the two groups is reported in Table S3 and the incidence of micro-hemorrhages is reported in Table S4.

**Table S3** Incidence of patchy pattern of atelectasis per anatomical region, comparison between the groups.

|  | Right lung | |  | Left lung | |  |
| --- | --- | --- | --- | --- | --- | --- |
|  | Continuous mode | 30:2 mode | p-value | Continuous mode | 30:2 mode | p-value |
| Upper ventral | 6/8 | 1/8 | 0.041 | 3/8 | 2/8 | 1.000 |
| Upper dorsal | 2/8 | 2/8 | 1.000 | 2/8 | 1/8 | 1.000 |
| Paracardiac | 3/8 | 0/8 | 0.200 | 1/8 | 0/8 | 1.000 |
| Lower ventral | 0/8 | 0/8 | N/A | 0/8 | 0/8 | 1.000 |
| Lower dorsal | 2/8 | 0/8 | 0.467 | 0/8 | 0/8 | 0.282 |

**Table S4** Incidence of microhemorrhages per anatomical region, comparison between the groups.

|  | Right lung | |  | Left lung | |  |
| --- | --- | --- | --- | --- | --- | --- |
|  | Continuous mode | 30:2 mode | p-value | Continuous mode | 30:2 mode | p-value |
| Upper ventral | 5/8 | 3/8 | 0.619 | 3/8 | 4/8 | 1.000 |
| Upper dorsal | 5/8 | 3/8 | 0.619 | 8/8 | 5/8 | 0.200 |
| Paracardiac | 5/8 | 3/8 | 0.619 | 5/8 | 2/8 | 0.315 |
| Lower ventral | 3/8 | 1/8 | 0.569 | 3/8 | 2/8 | 1.000 |
| Lower dorsal | 3/8 | 2/8 | 1.000 | 4/8 | 1/8 | 0.282 |

## Wet-dry ratio.

For each subject, 10 samples were taken from the same anatomical regions as the histopathological samples (5 samples per lung), to perform the wet-dry weight calculation, a method to assess edema in tissues.

The samples were placed on a metallic foil and weighted fresh, then left in an oven (37° C) for a week and re-weighted. The fraction of tissue left after the drying (wet-dry weight - expressed in grams) was used for a group comparison.

The results of the wet-dry weight are reported in Figure 2A.

*
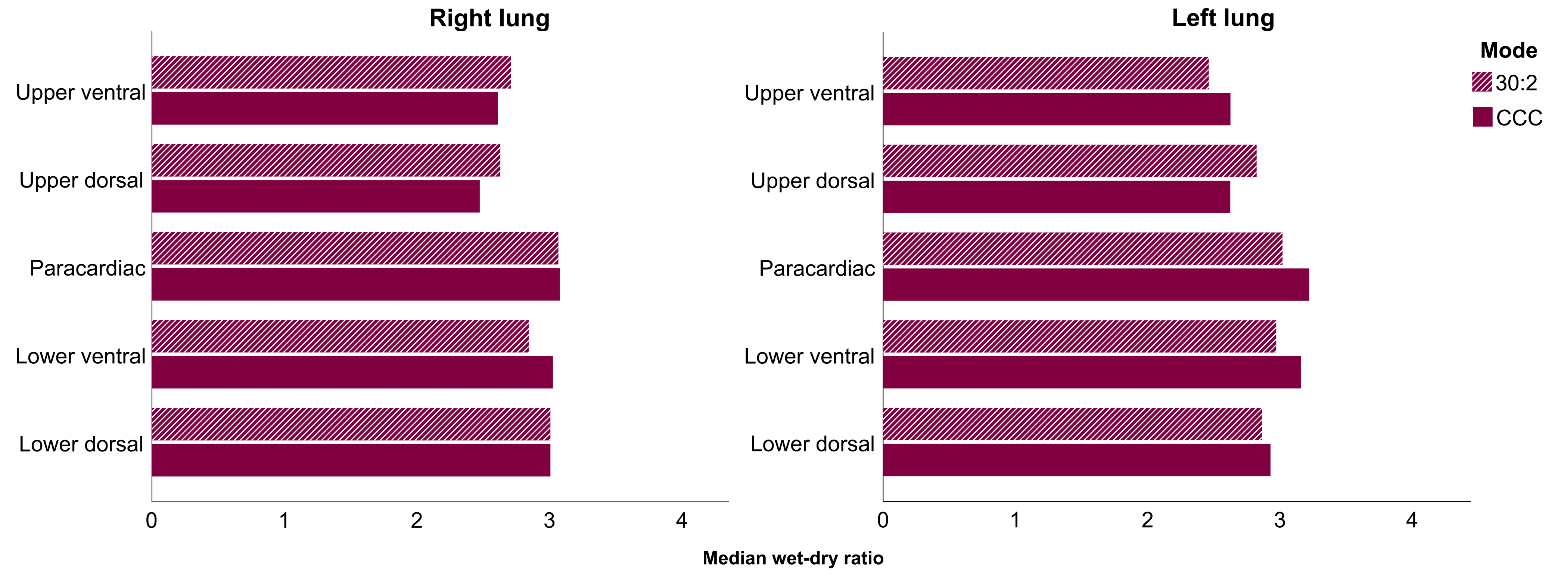
*

***Fig. S2*** *Comparison of Wet-dry ratio per anatomical region and CPR mode.*
