# Supplementary material for: Continuous chest compressions are associated with higher peak inspiratory pressures when compared to 30:2 in an experimental cardiac arrest model
Source: Intensive Care Med Exp. 2023 Nov 8;11:75. doi: 10.1186/s40635-023-00559-7 (PMC10632261; doi:10.1186/s40635-023-00559-7)
Supplement: Supplementary file 5 — Additional file 5. Histopathological analysis. [file 40635_2023_559_MOESM5_ESM.pdf]

Individual and material: Stryker, Pig 1, lung tissue.

Results: Sample identification and histopathology findings are shown in the table below.

**Stryker section code**	**SVA section code**	**Atelectasis**	**Oedema**	**Signs of hyperinflation**	**Other findings having potential interest**
Right upper ventral	LDJ-682/22	About 70% multifocal	+ subpleural areas, interlobular septa	<10%	Recent hemorrhages, multifocal interstitial inflammation
Right upper dorsal	LDJ-683/22	About 70% lobular	0	<10%	Recent hemorrhages, multifocal interstitial inflammation
Right lower ventral	LDJ-684/22	>90%	+ interlobular septa	<5%	Interstitial leukocytes
Right lower dorsal	LDJ-685/22	>90%	+ subpleural	<5%	Interstitial leukocytes
Right paracardiac	LDJ-686/22	>90%	0	0	Interstitial leukocytes
Left upper ventral	LDJ-687/22	About 70% multifocal, most prevalent in peribronchial areas	0	About 20%	Interstitial leukocytes
Left upper dorsal	LDJ-688/22	About 80%, several lobules	++++ subpleural, proteinaceous fluid in alveoli	<10%	Subpleural hemorrhages, Multifocal/lobular interstitial inflammation
Left lower ventral	LDJ-689/22	>90%	0	<5% in residual alveoli	Interstitial leukocytes
Left lower dorsal	LDJ-690/22	>90%	0	<5% in residual alveoli	Interstitial leukocytes
Left paracardiac	LDJ-691/22	About 90%	++ subpleural	About 10%	Subpleural hemorrhages, interstitial leukocytes

Table headings

Atelectasia (%): Histological sections were examined using a low-power objective (x2). The area of the lung showing collapsed alveoli was scored in a semiquantitative, subjective way as % of the total section area. The pattern of atelactasia was defined as focal/multifocal (patchy) or diffuse (when the whole section or vast parts of it showed collapsed alveoli (i.e., 80% or more) the word diffuse was omitted. Some sections showed atelectasia predominantly in certain areas, like subpleural areas (i.e., pig nr 3, LDJ-708/22), peribronchial areas, or along interlobular septa, the alveolar collapse also could affect entire lung lobules.

Signs of hyperinflation (%): The areas showing overdistended alveoli and confluent alveoli were scored in a subjective way similar to atelectasia.

Oedema: A sign of oedema was the presence of proteinaceous slightly eosinophilic or basophilic homogeneous material (i.e., fluid) in open alveoli, but this was also seen in inflamed lungs or in areas of haemorrhage (i.e., pig nr 4, LDJ-715, 718, 720/22). The presence of fluid was most difficult to evaluate in atelectatic lungs. Here, a feature considered as indicative of oedema was dilated lymphatic vessels in the pleura and the subpleural areas or in the interlobular septa.
Oedema was scored as +++++: very severe and extensive, ++++: severe and extensive, +++: severe, ++: moderate, +: mild, 0: significant lesions not observed.

Other findings

Fresh hemorrhages (i.e., having recent origin) were found in many lungs.
Leukocyte infiltrates: Inflammatory cell infiltrates varied in intensity between the animals and sections, consisting predominantly of macrophages and lymphocytes, but occasional lungs also displayed polymorphs. Macrophages and neutrophils are considered as acute reaction cells, but lymphocytes are chronic cells that could have been already in the lungs before the experiments commenced, at least part of the lymphocytes. The sections examined did not show microorganisms or foreign particles that could have caused the cell reactions observed.
The leukocytes were seen most frequently in the perivascular and the peribronchial areas (i.e., pig nr 8, L-754/22), the interlobular septa and the subpleural areas. (i.e., pig nr 18, LDJ-857/22. Leukocytes also were observed in the alveolar septa, and some lungs showed leukocytes free in the alveolar lumens (pig nr 4, LDJ-715/22). Macrophages were the predominant cells in the alveoli, less commonly also granulocytes were observed (pig nr 9, LDJ-762/22). In some sections, scattered mitotic figures were noticed among the inflammatory cells free in the alveoli (i.e., pig nr 4, LDJ-715, 716/22).
The histological diagnosis of inflammation in the material examined was based on the occurrence of leukocytes. When leukocytes in the sections were numerous (i.e., pig nr 2, LDJ-701/22, pig nr 4, LDJ-715/22) the diagnosis of inflammation was straightforward, but many lungs showed low numbers of leukocytes, which was not necessarily consistent with an on-going inflammatory reaction. Hence, since the threshold between the cell infiltrates and an inflammation was not sharp, to decide when low numbers of infiltrated leukocytes were considered as an inflammatory reaction sometimes may have been an arbitrary decision (i.e., pig nr 3, LDJ-702/22), especially in atelectatic lungs.
Occasional sections displayed a few other **uncommon findings**, like numerous small-sized vessels having thickened tunica media, or fibrosis, in the pleura and subpleural areas (pig nr 5, LDJ-730/22.
Pig nr 9, LDJ-730/22, showed many alveolar cells having enlarged, vesicular nucleus (it could be an artifact, but the reason for this is unknown).
