# Supplementary material for: Continuous chest compressions are associated with higher peak inspiratory pressures when compared to 30:2 in an experimental cardiac arrest model
Source: Intensive Care Med Exp. 2023 Nov 8;11:75. doi: 10.1186/s40635-023-00559-7 (PMC10632261; doi:10.1186/s40635-023-00559-7)

Pig 1
CCC mode

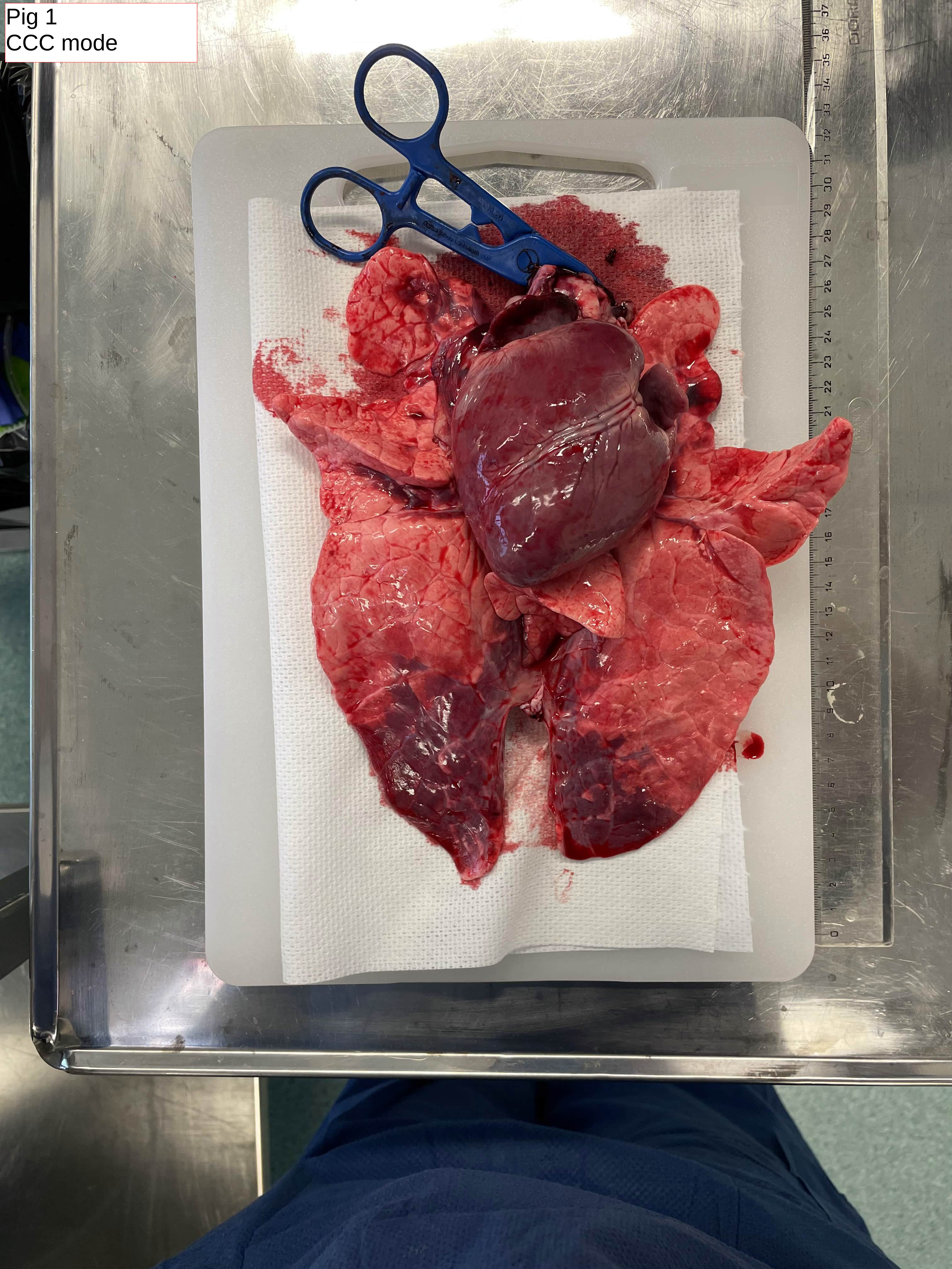

Pig 1
CCC mode

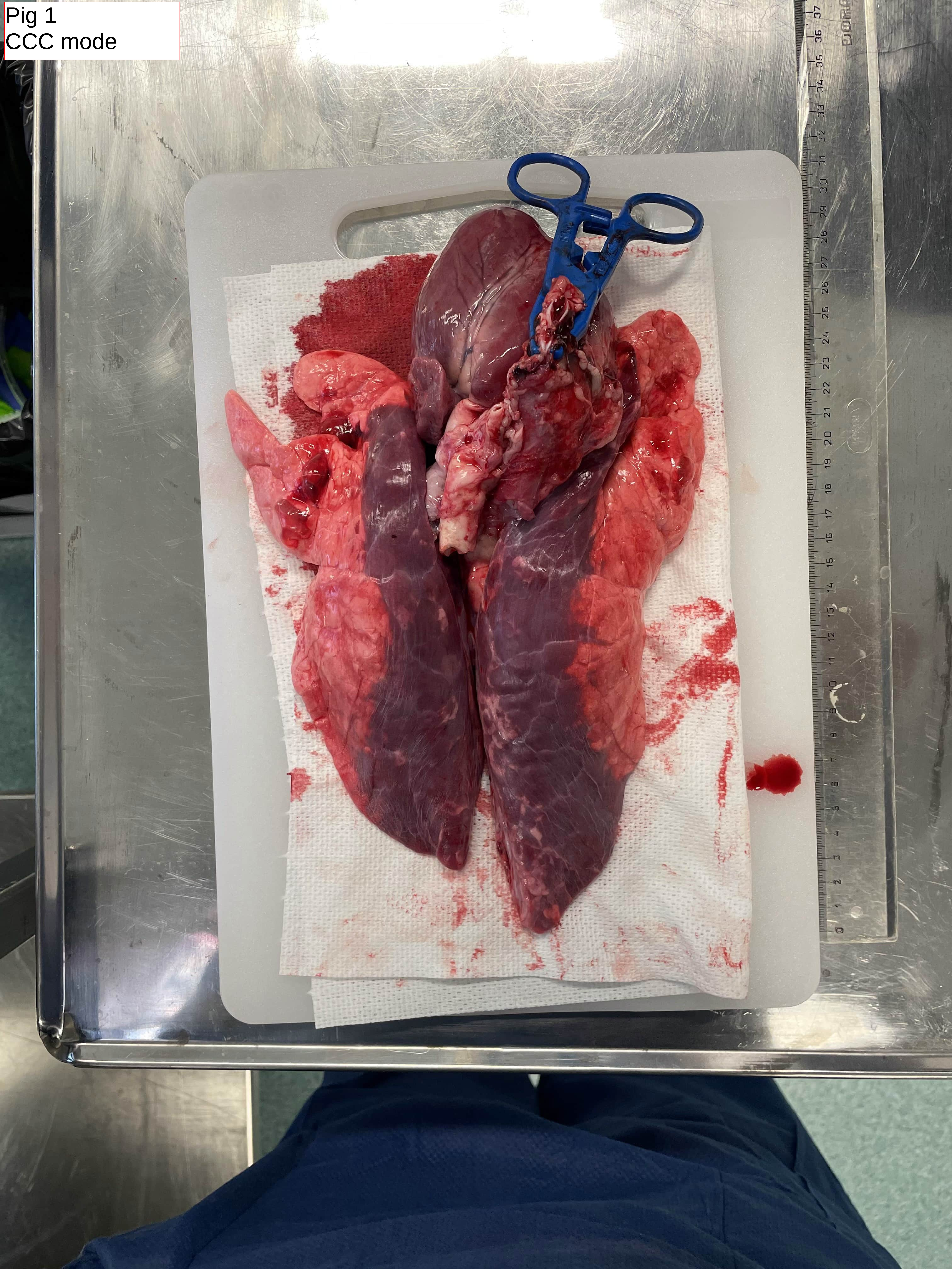

Pig 2
CCC mode

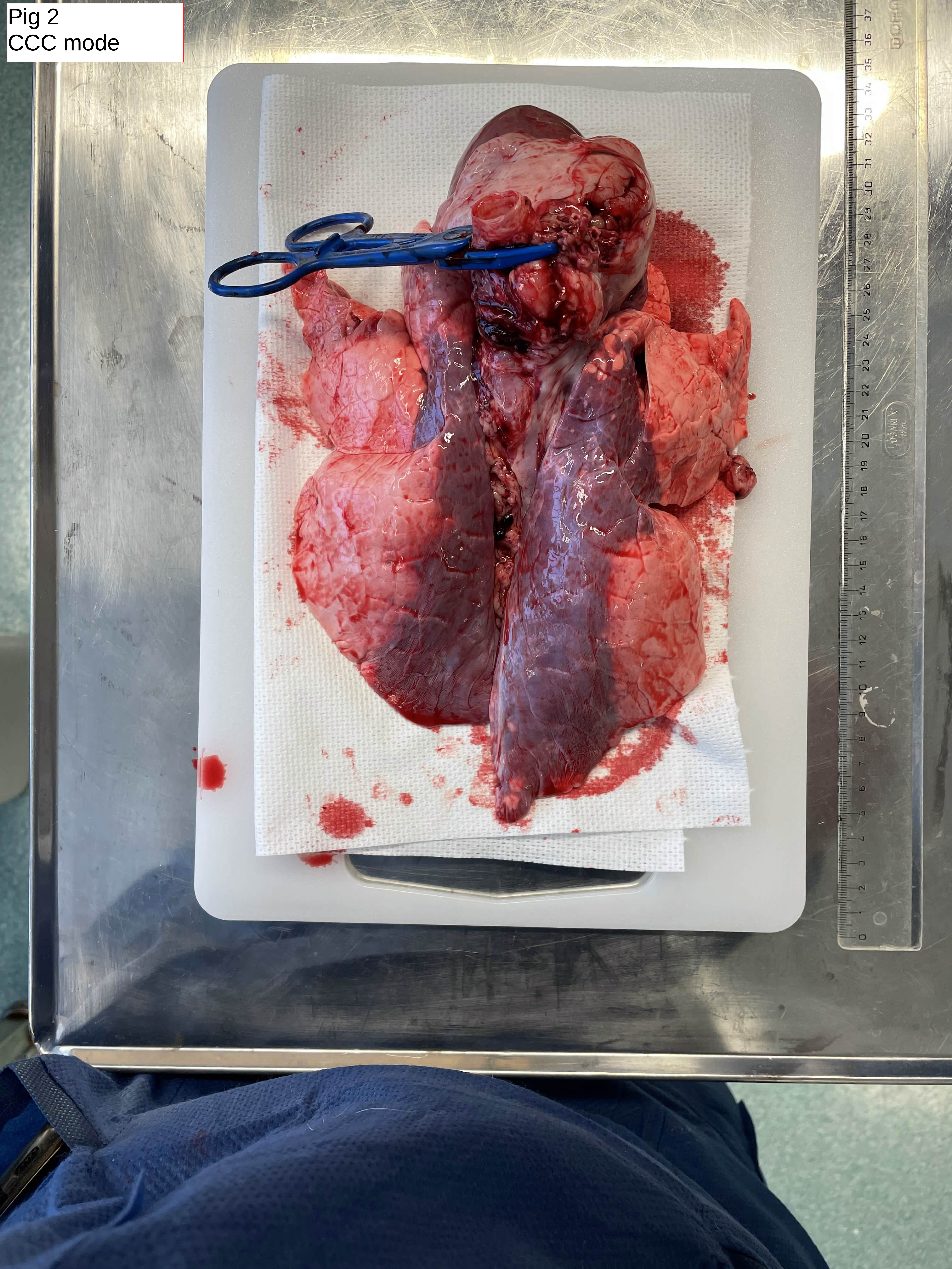

Pig 2
CCC mode

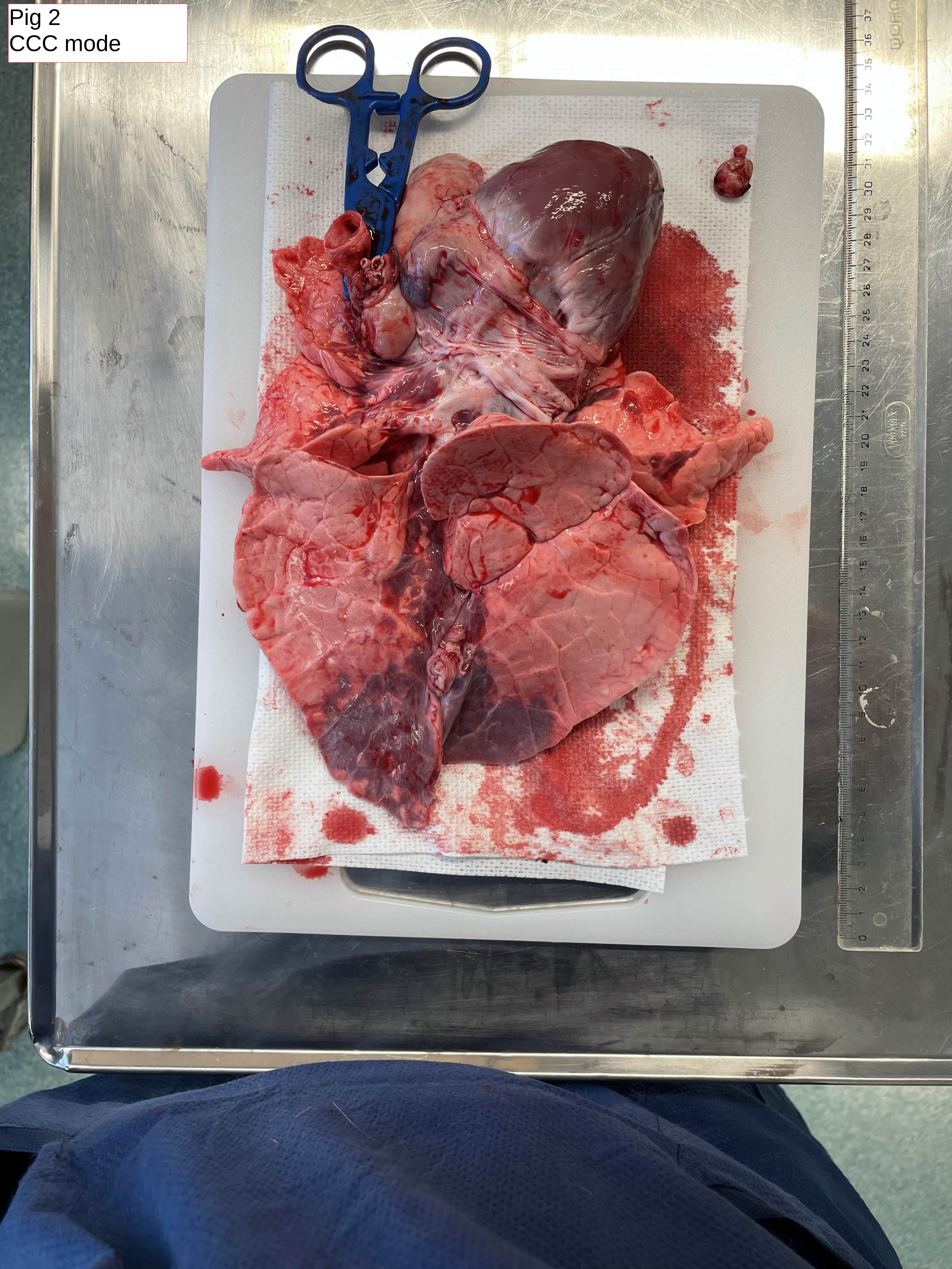

Pig 5
30:2 mode

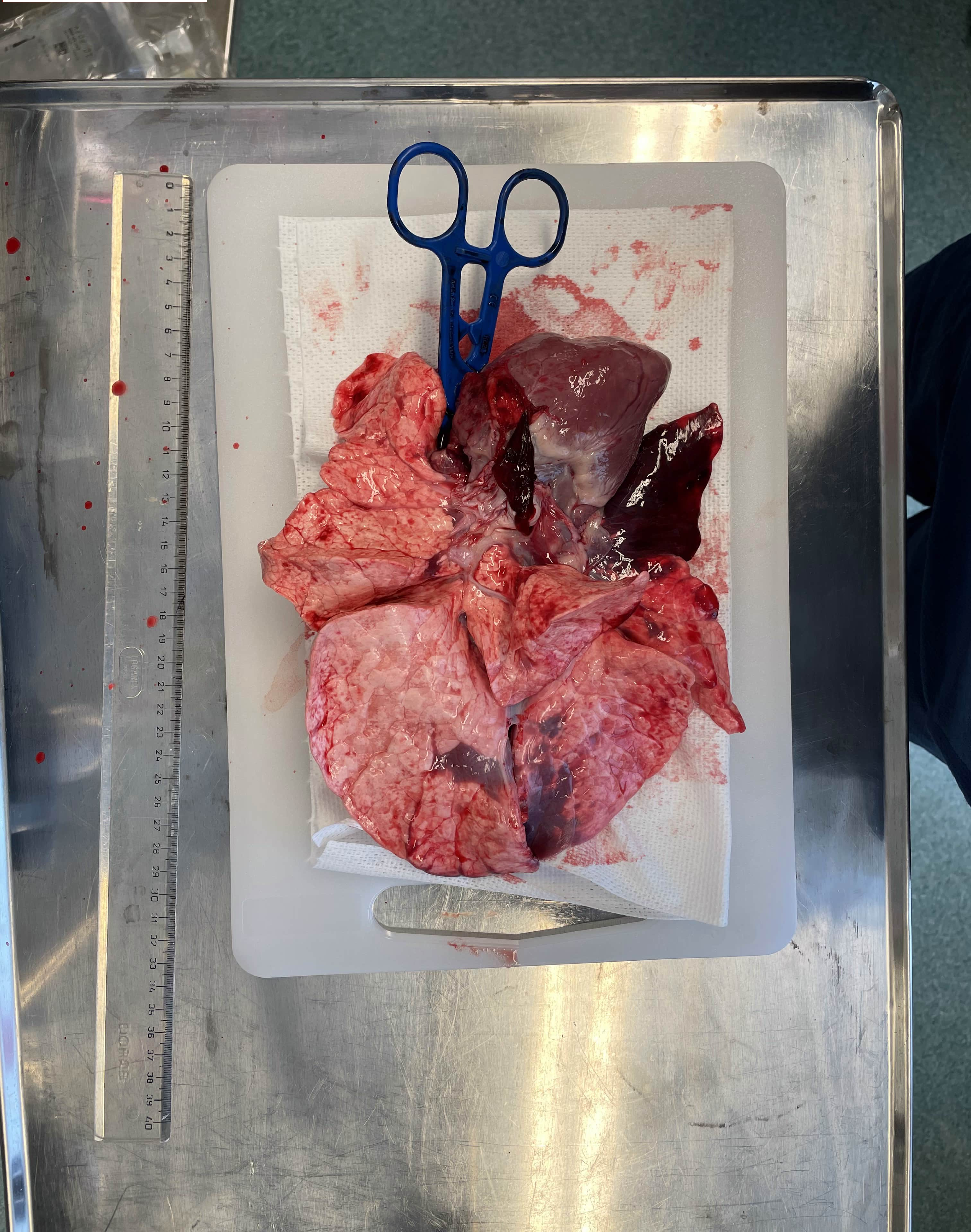

Pig 5
30:2 mode

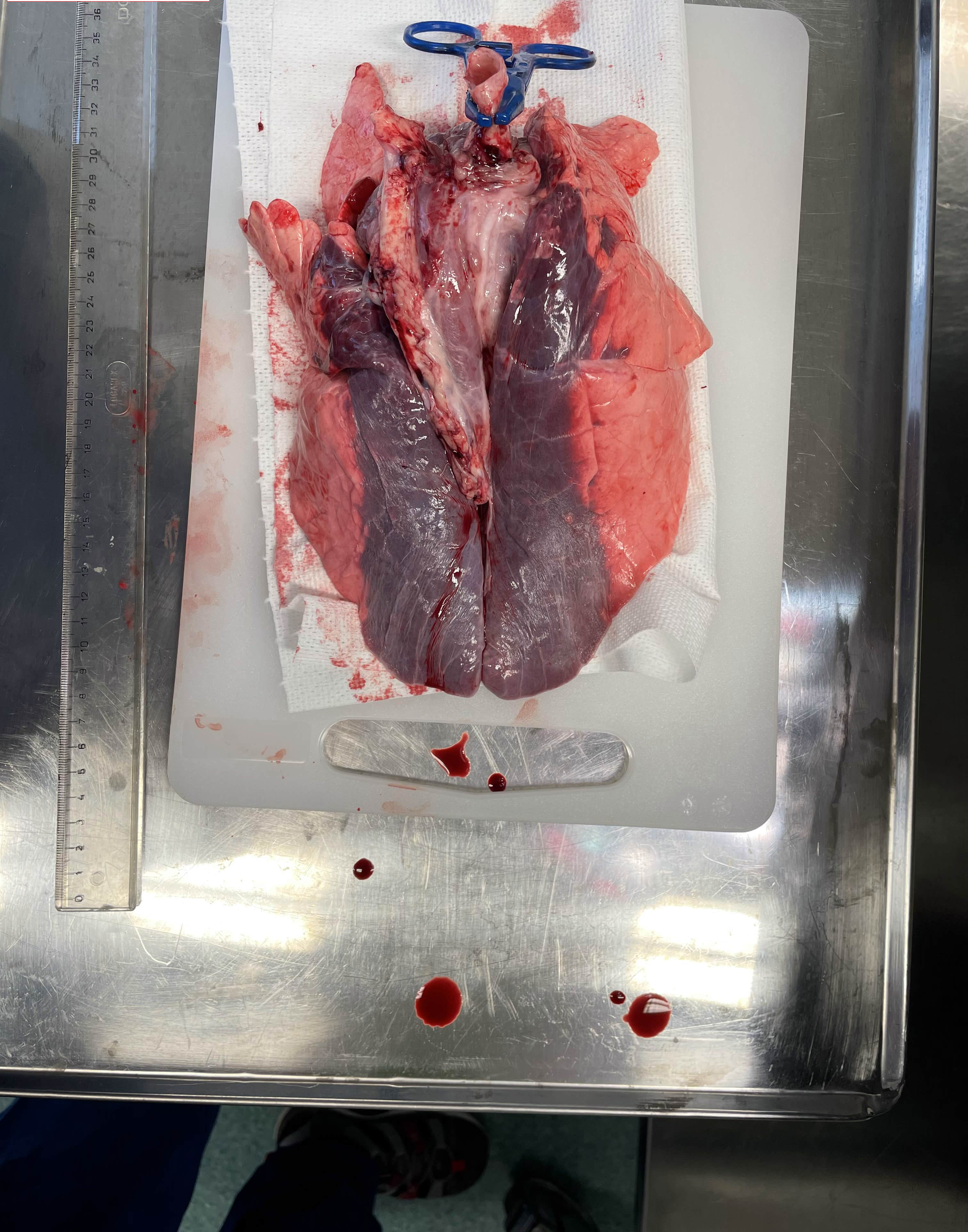

Pig 6
30:2 mode

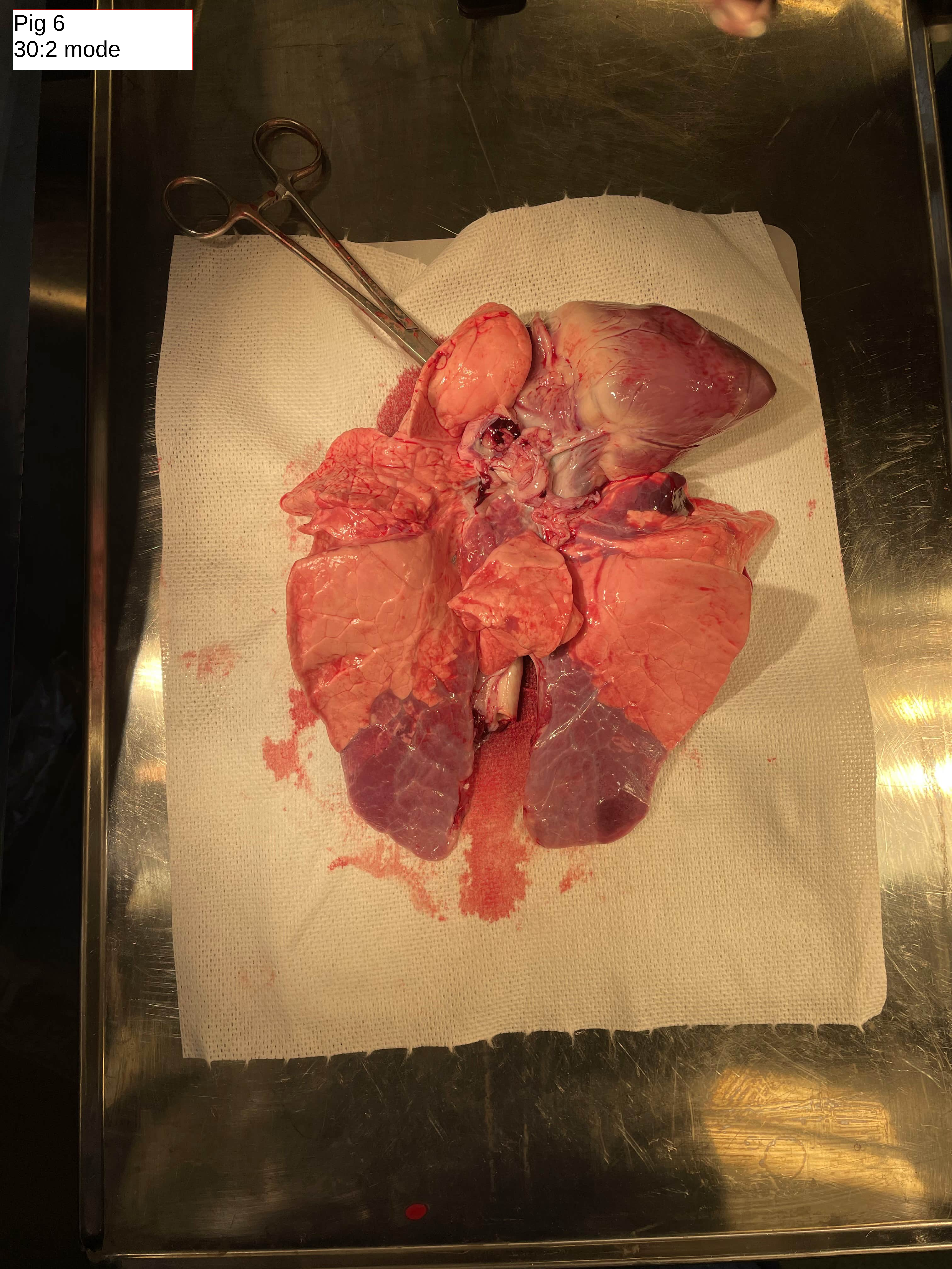

Pig 6
30:2 mode

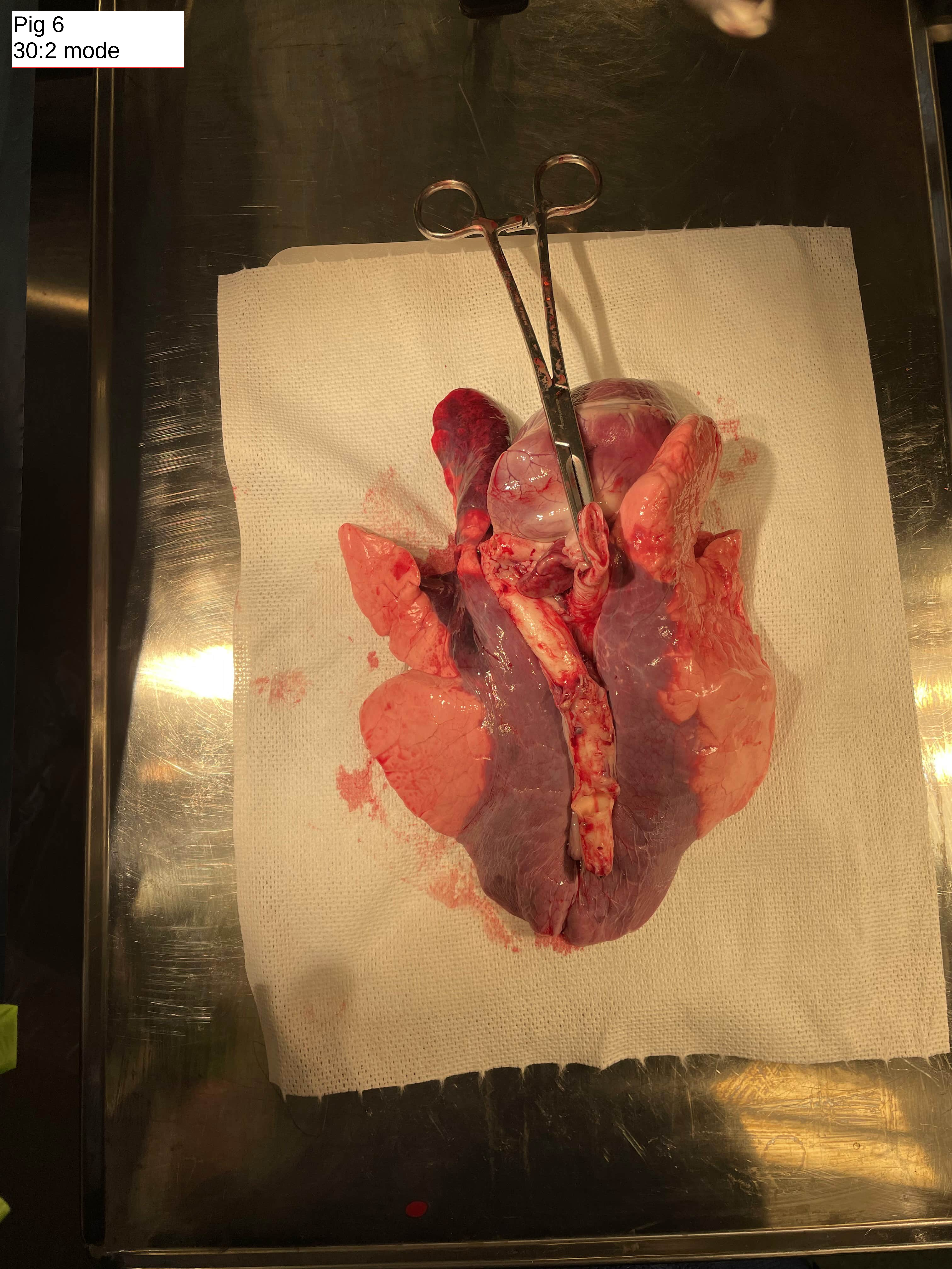

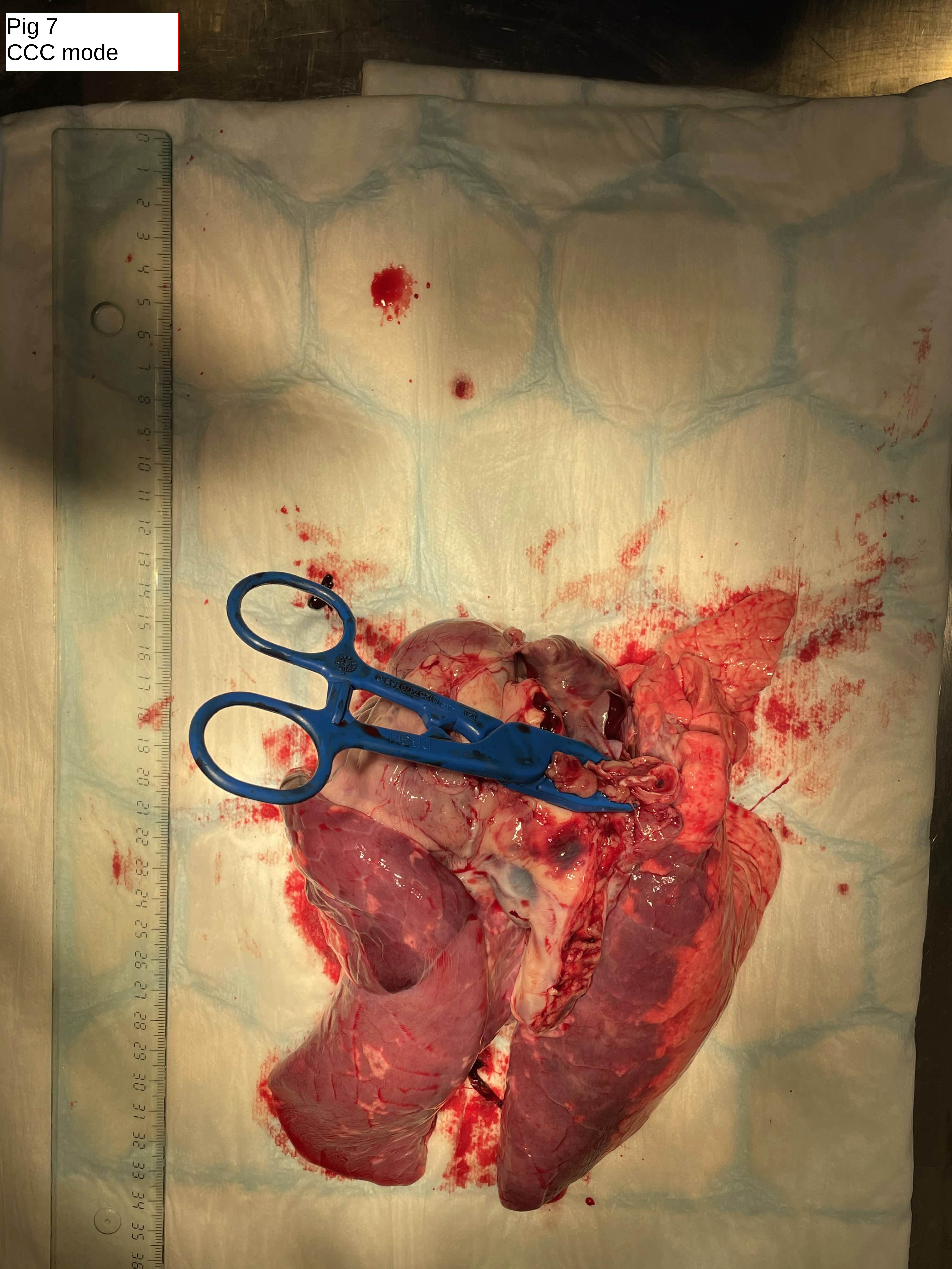

Pig 7
CCC mode

Pig 8
CCC mode

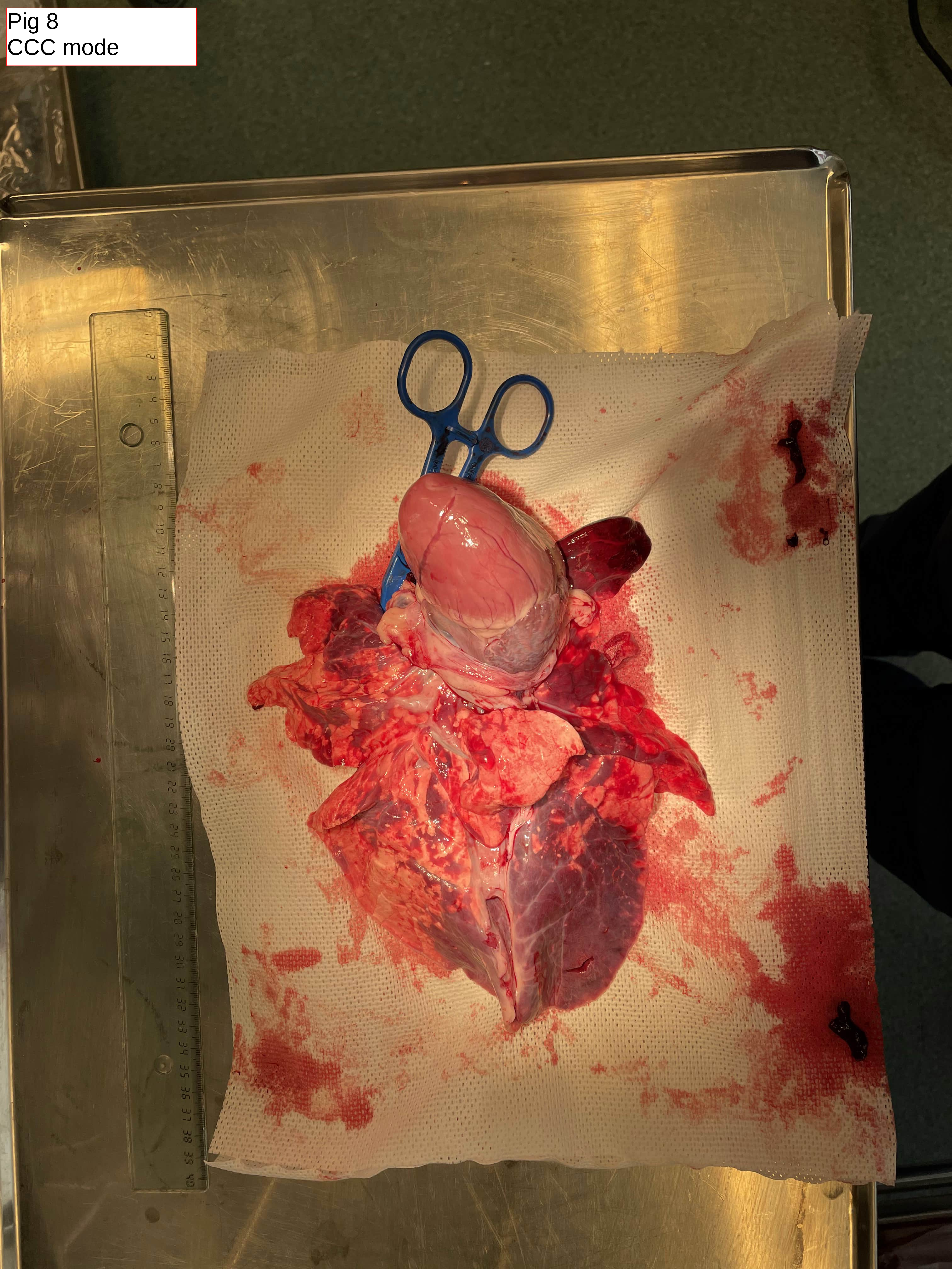

Pig 8
CCC mode

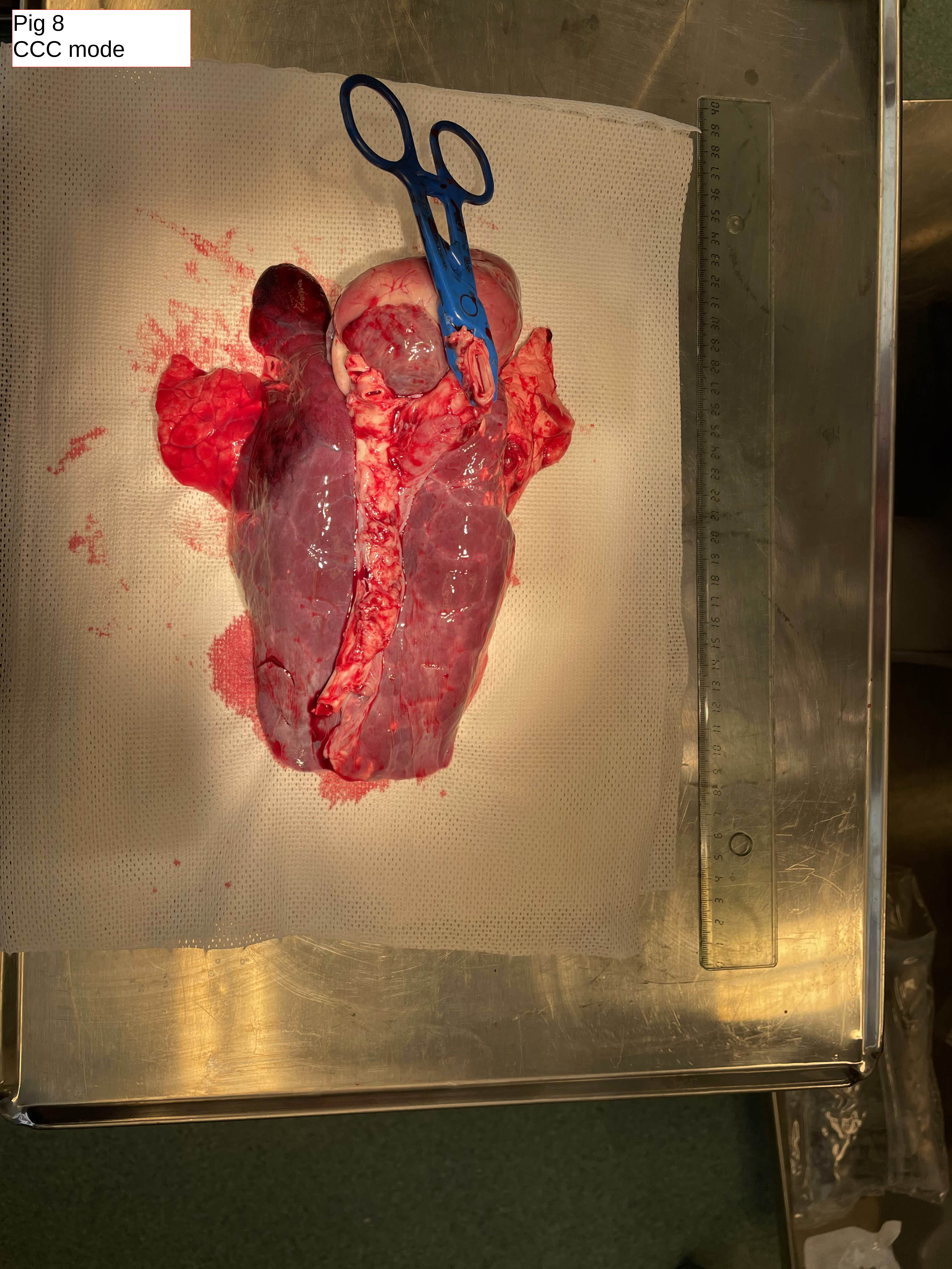

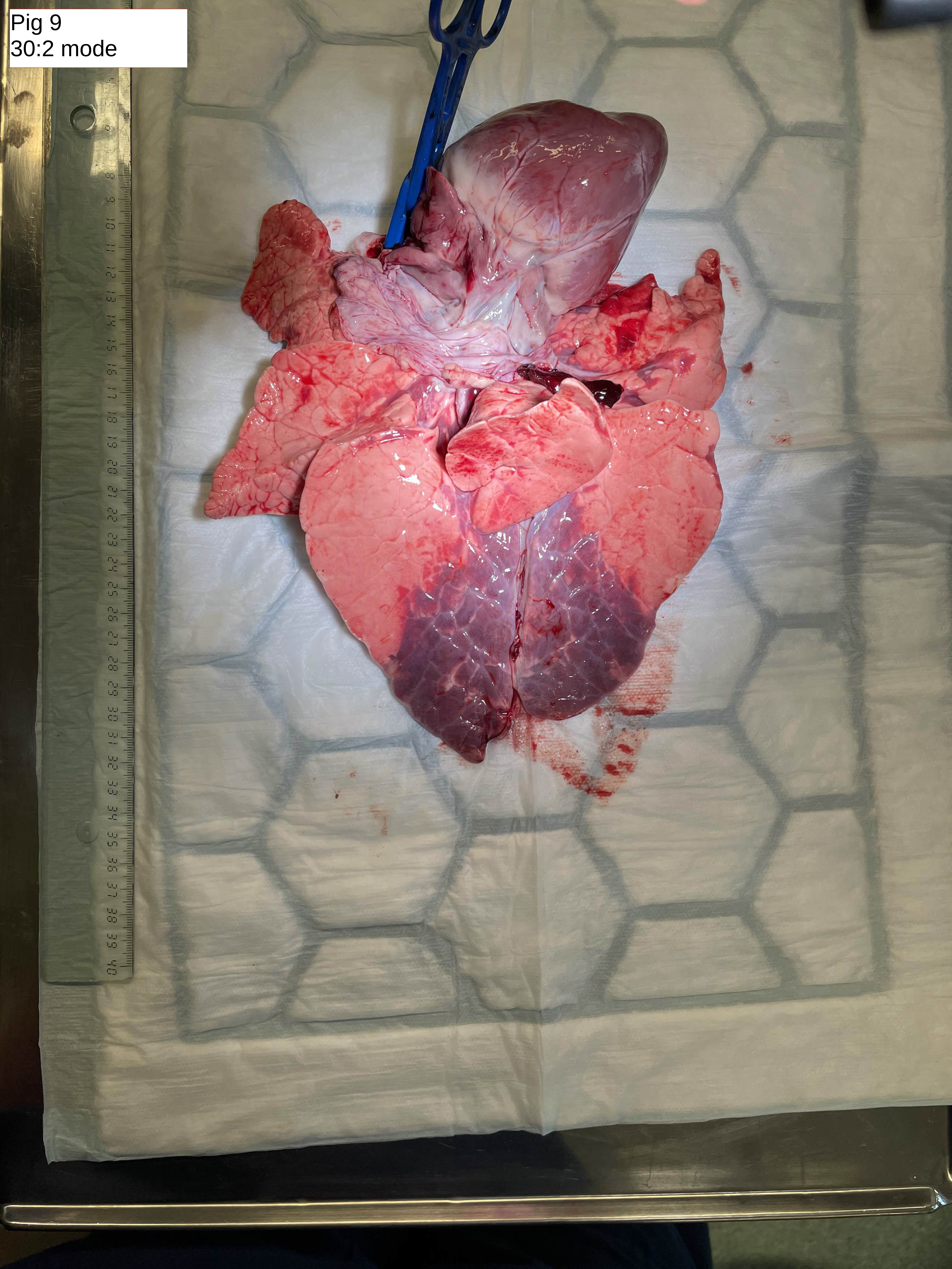

Pig 9
30:2 mode

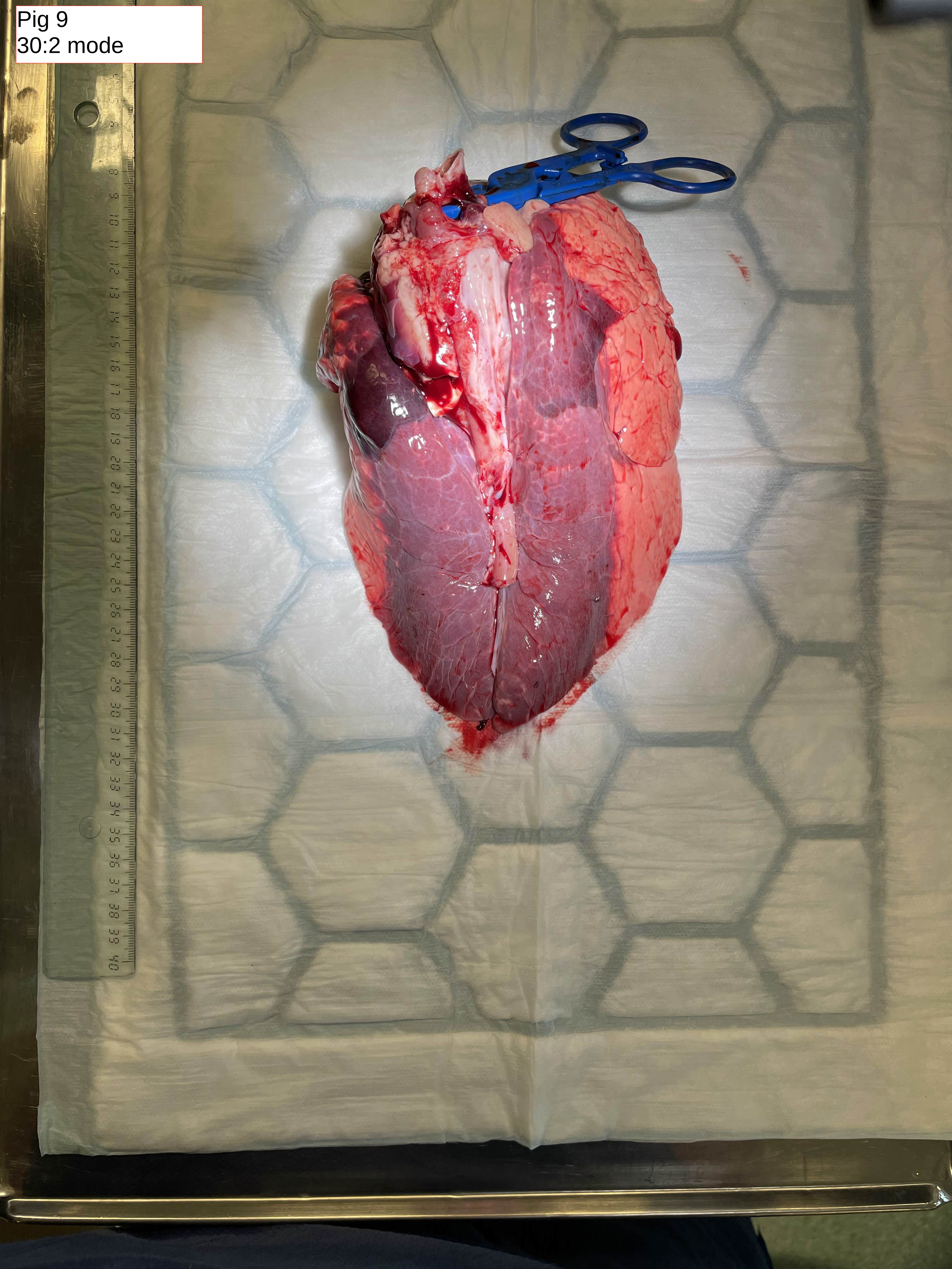
Pig 9
30:2 mode

Pig 10
30:2 mode

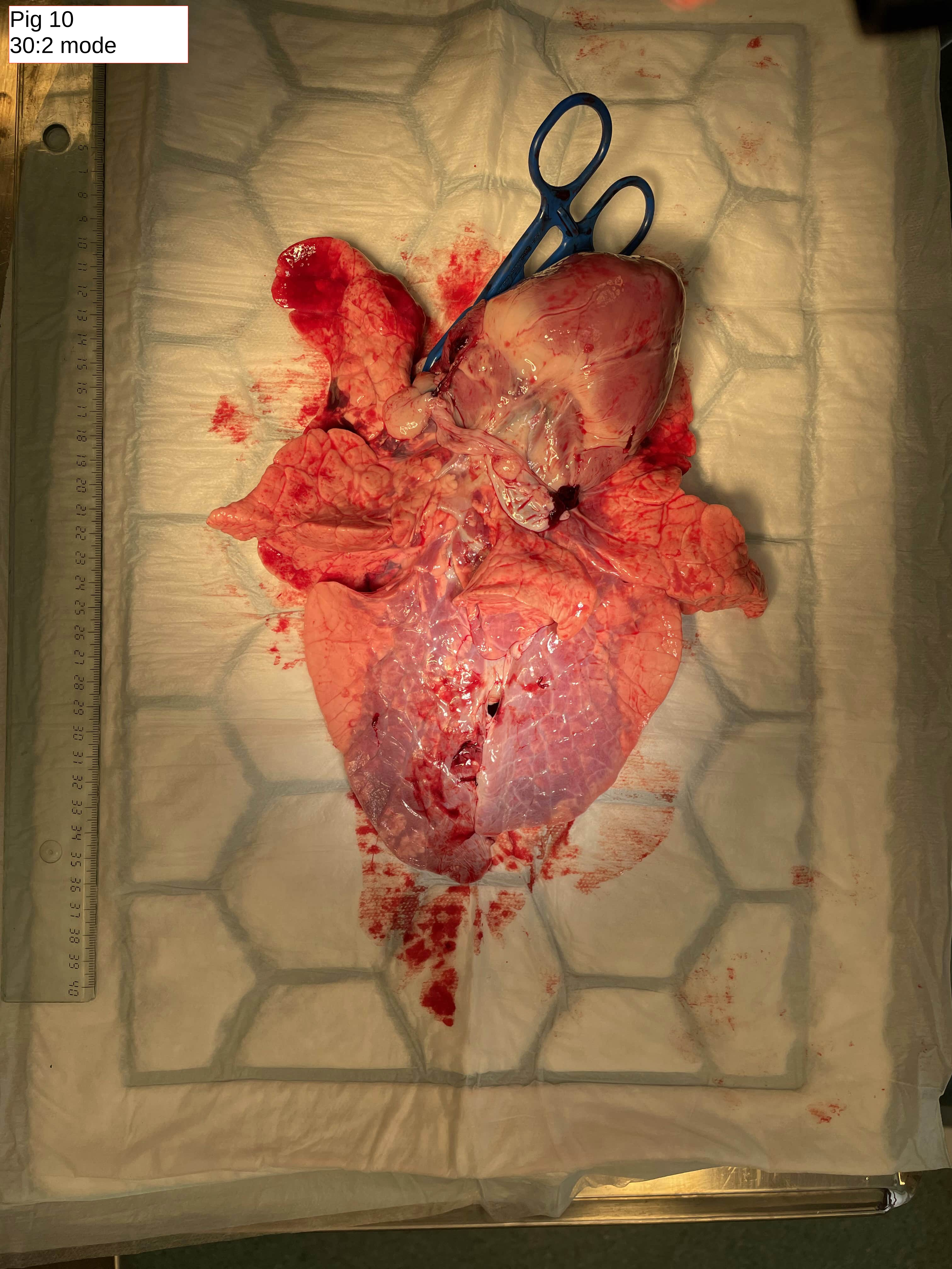

Pig 10
30:2 mode

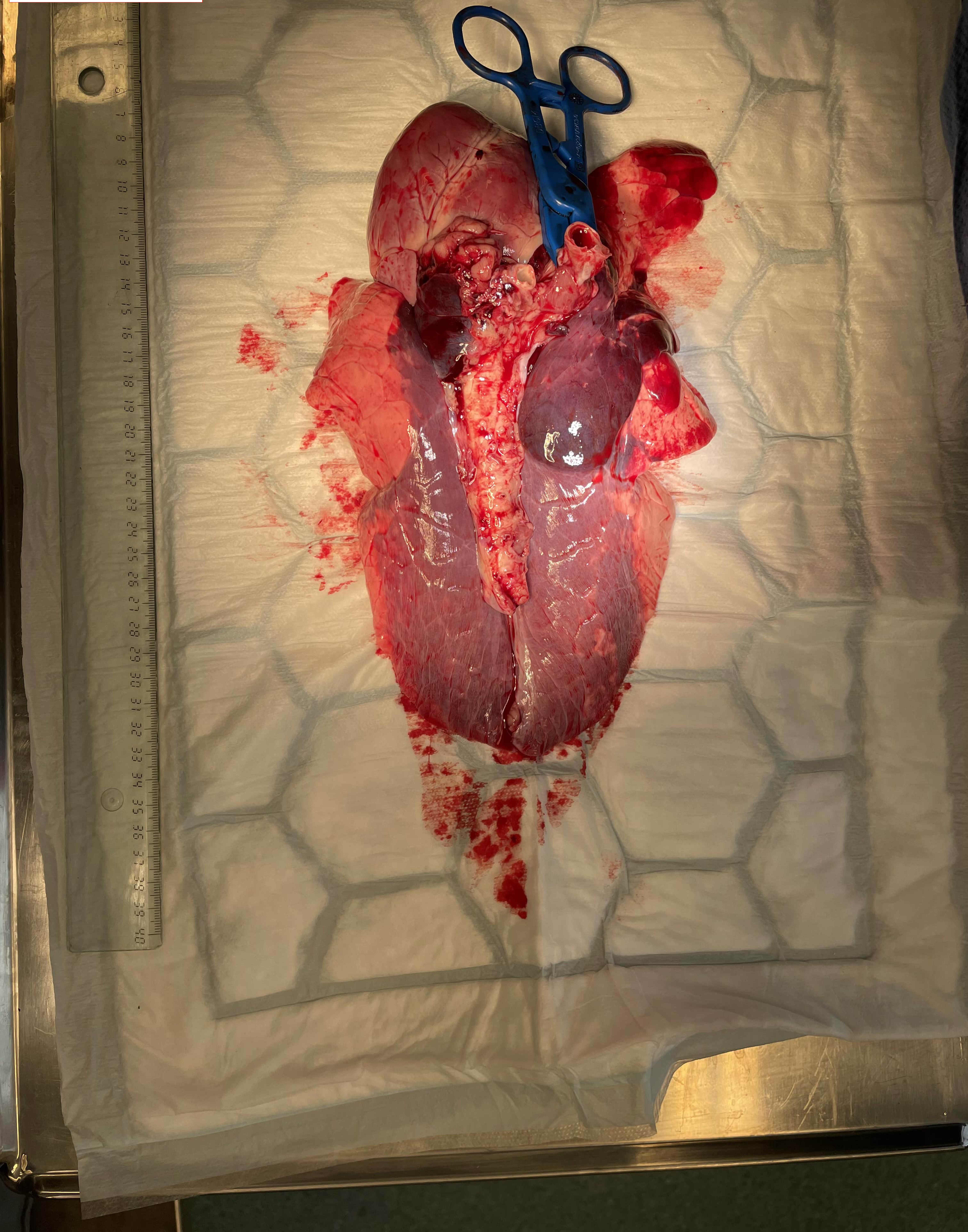

Pig 11
30:2 mode

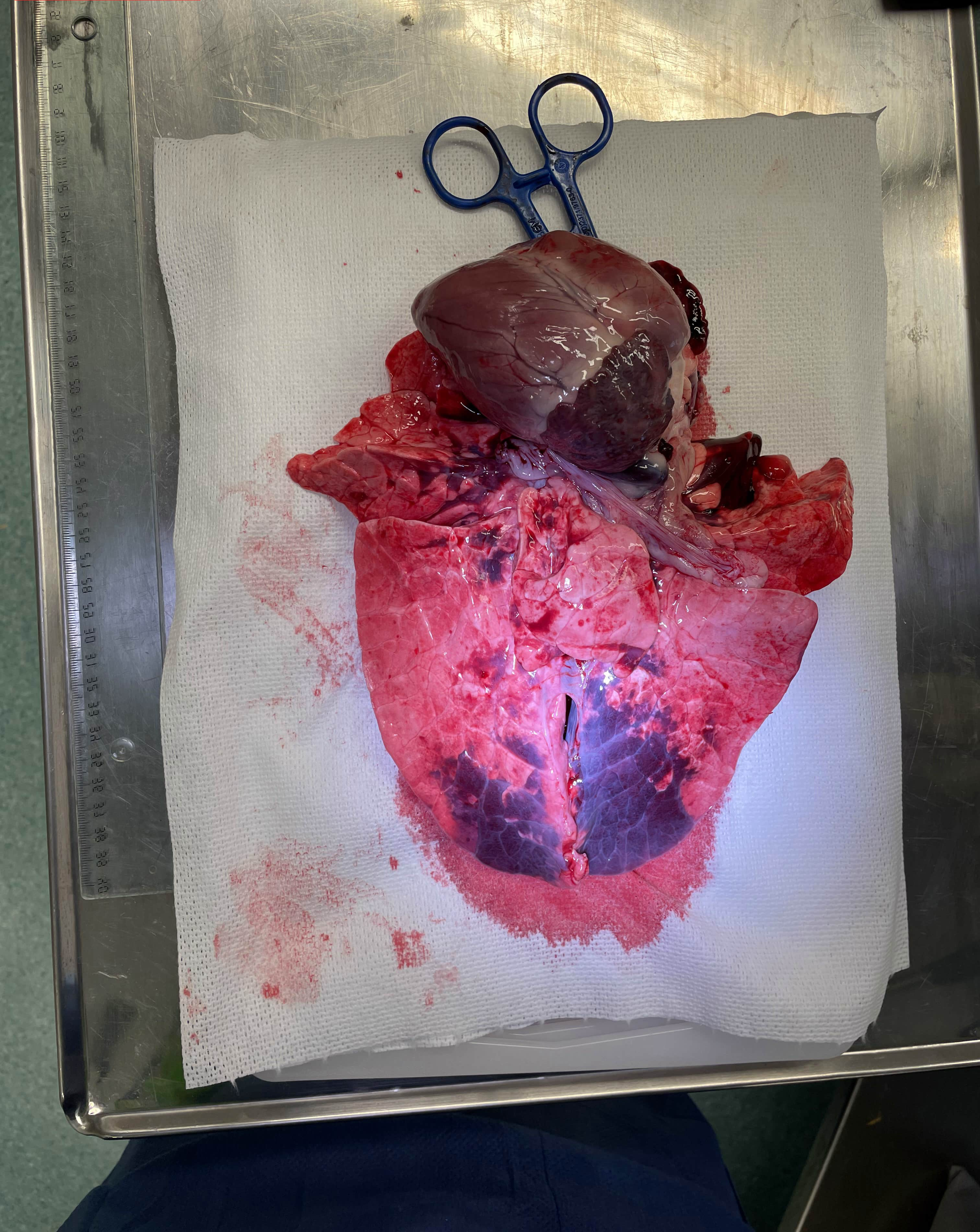

Pig 11
30:2 mode

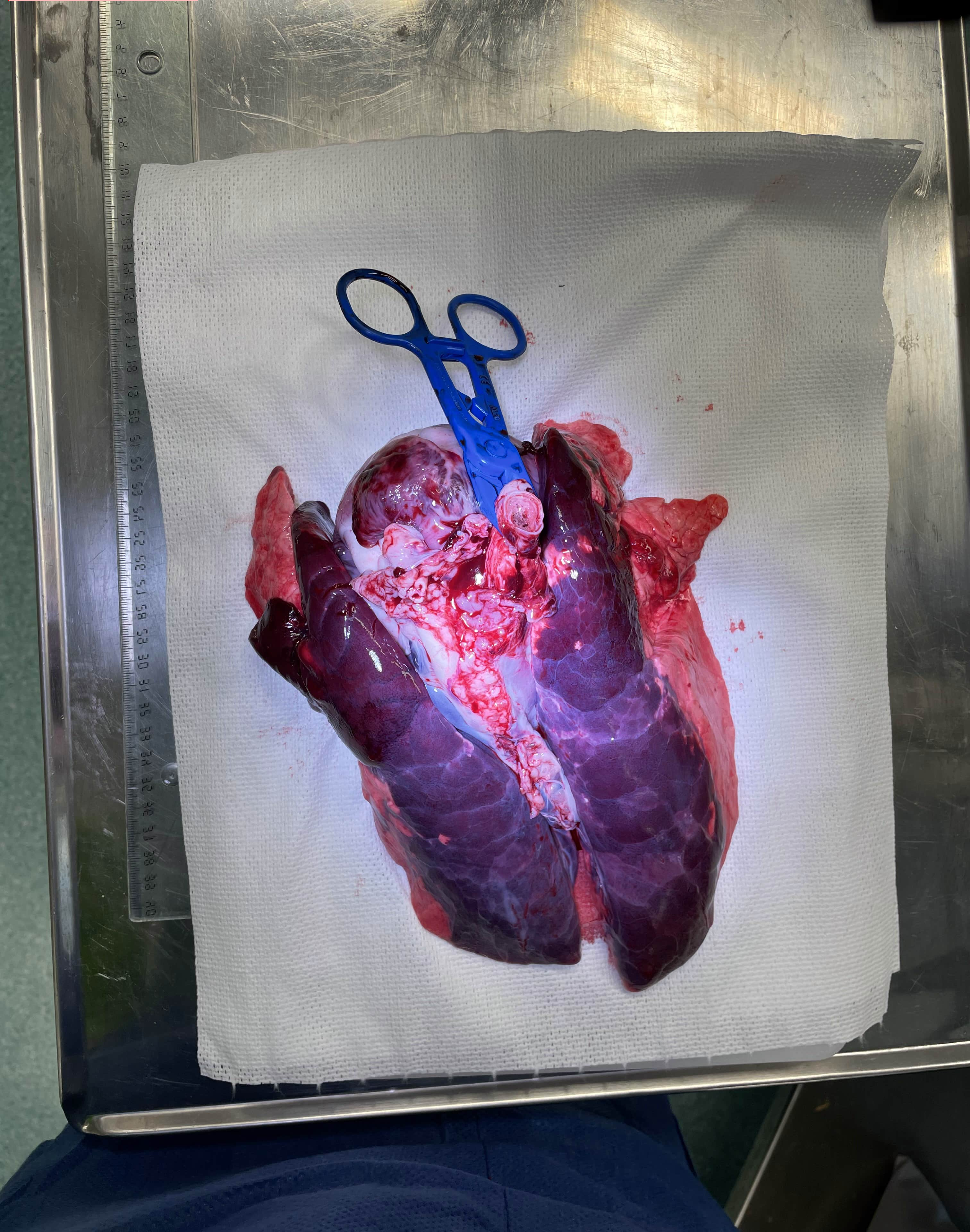

Pig 12
CCC mode

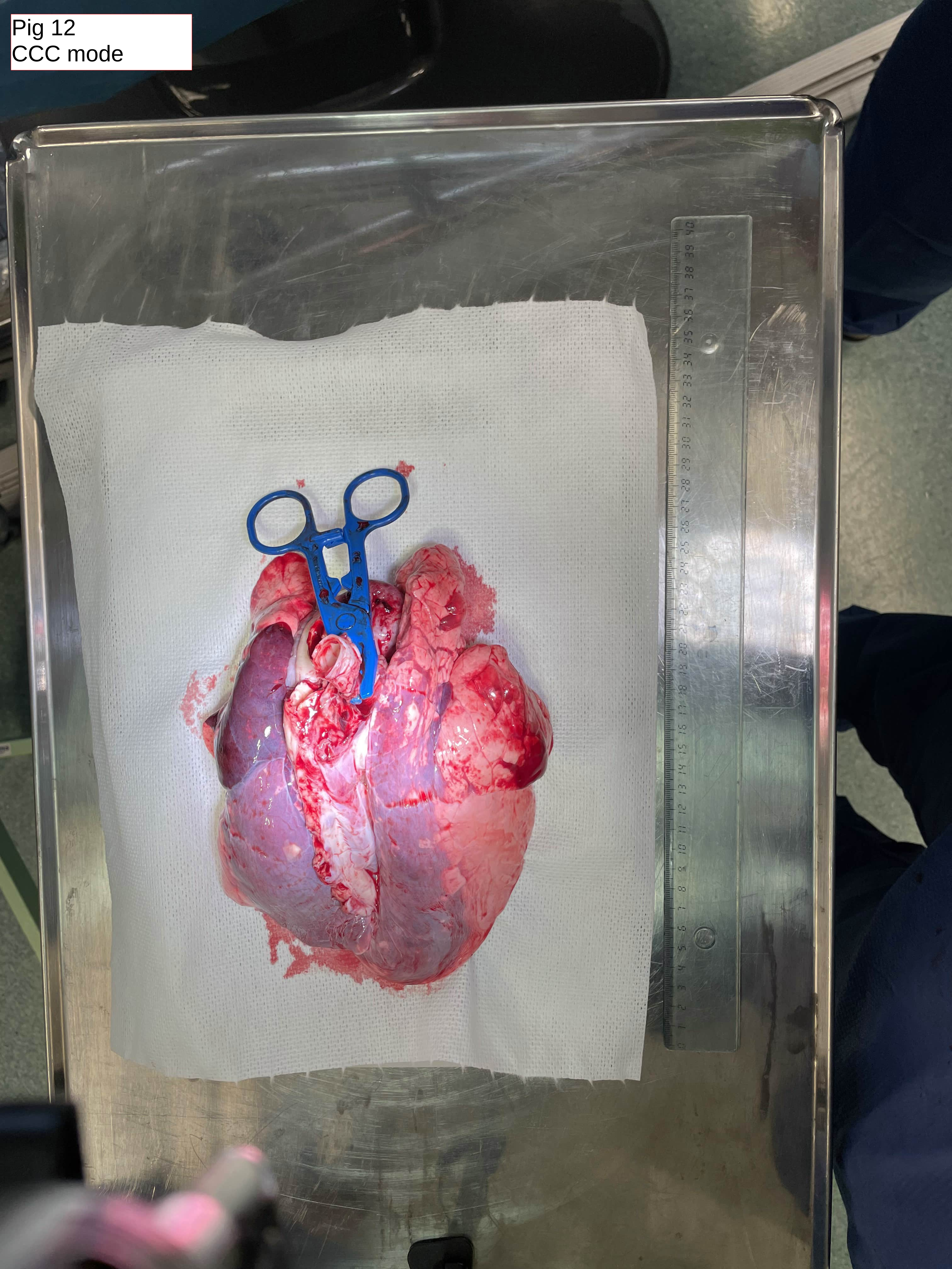

Pig 12
CCC mode

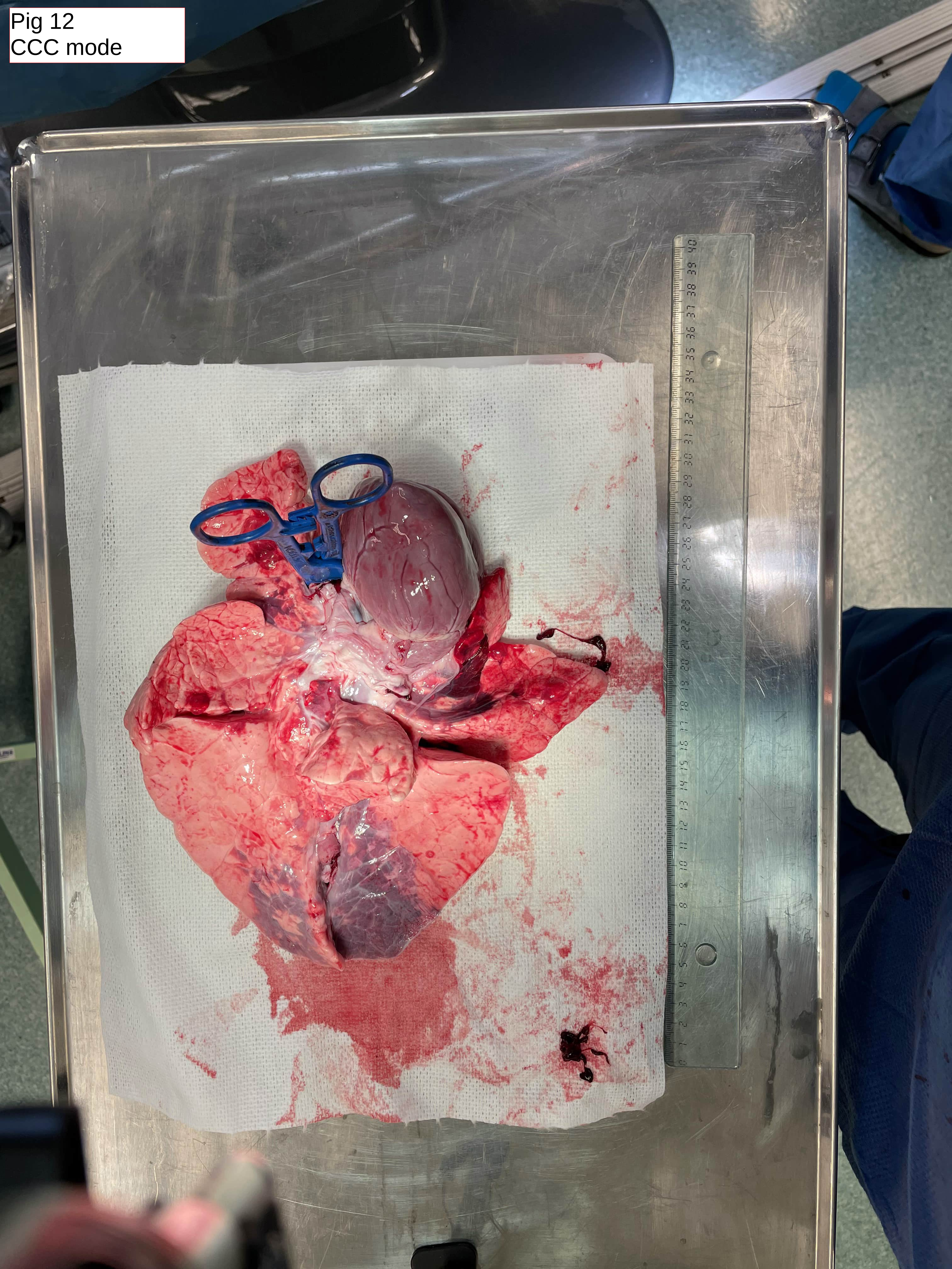

Pig 13
CCC mode

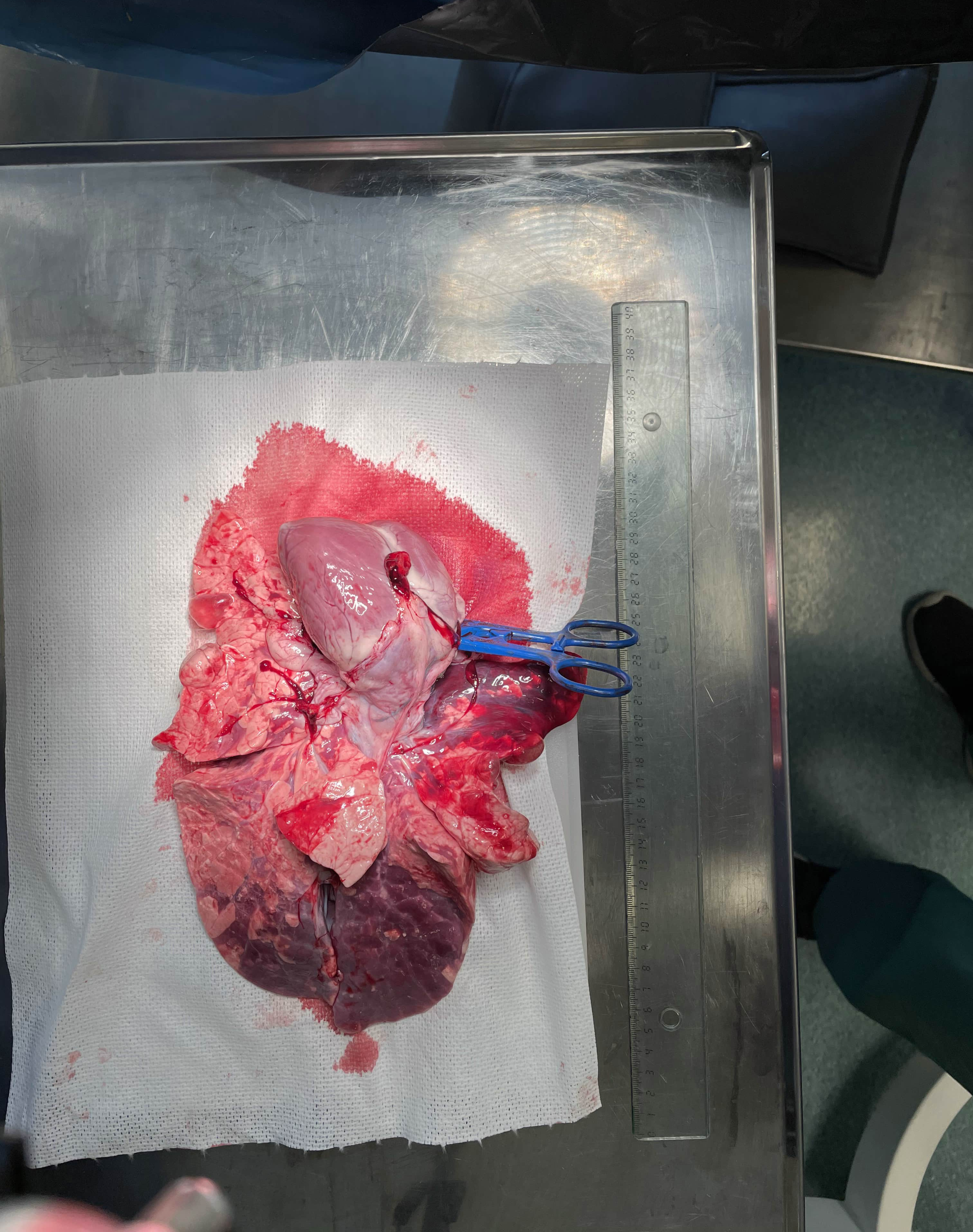

Pig 14
CCC mode

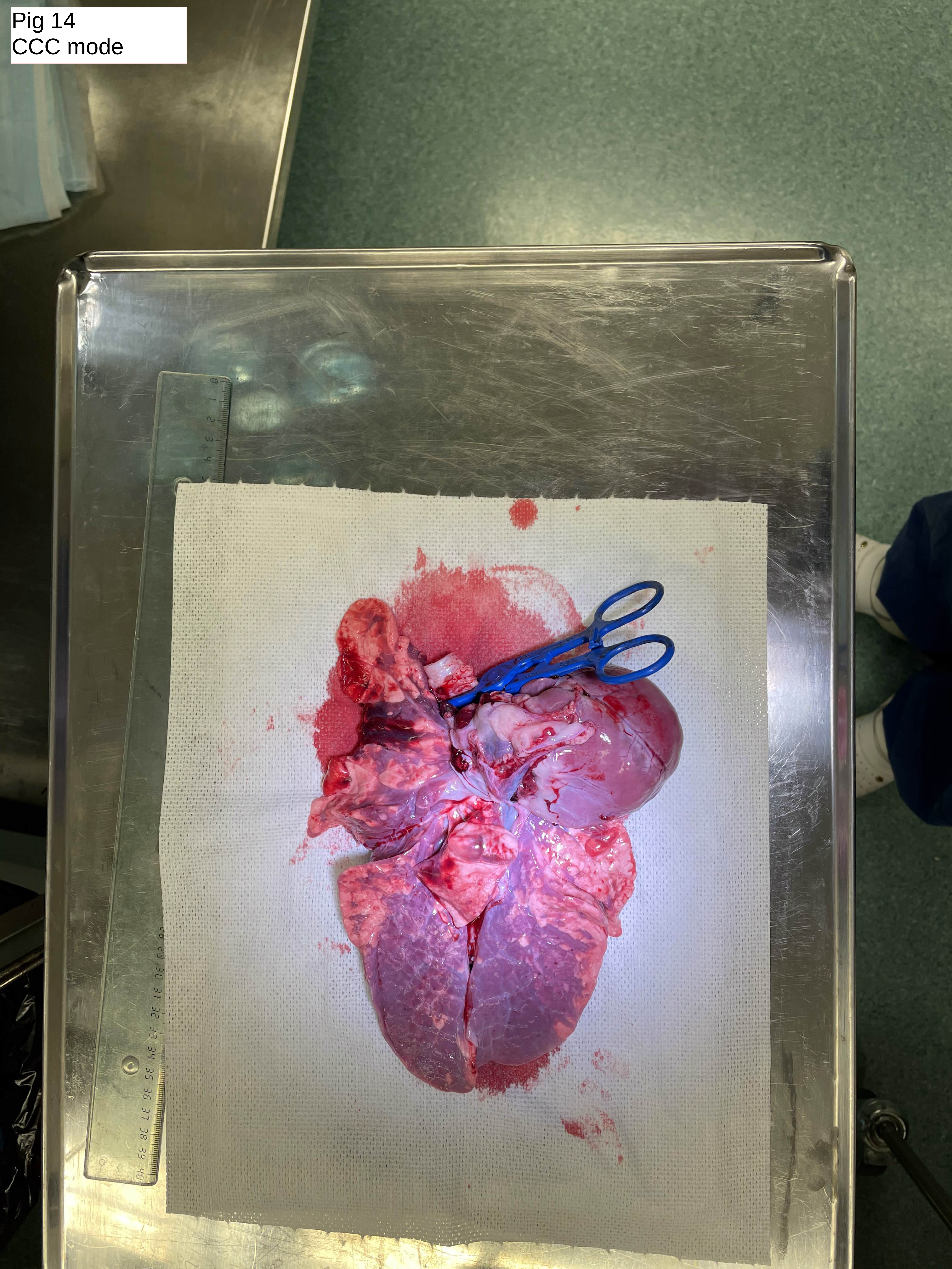

Pig 14
CCC mode

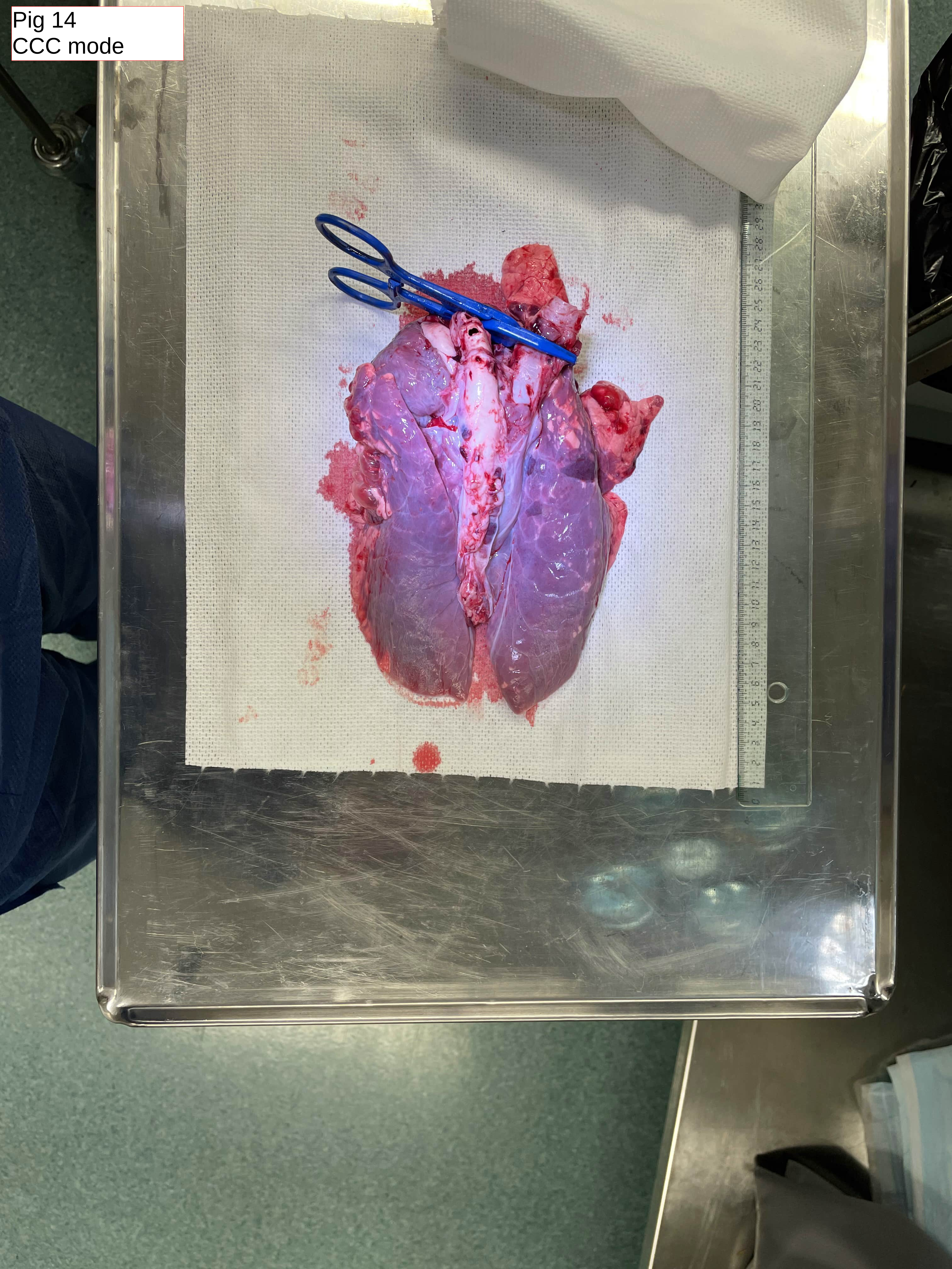

Pig 15
30:2 mode

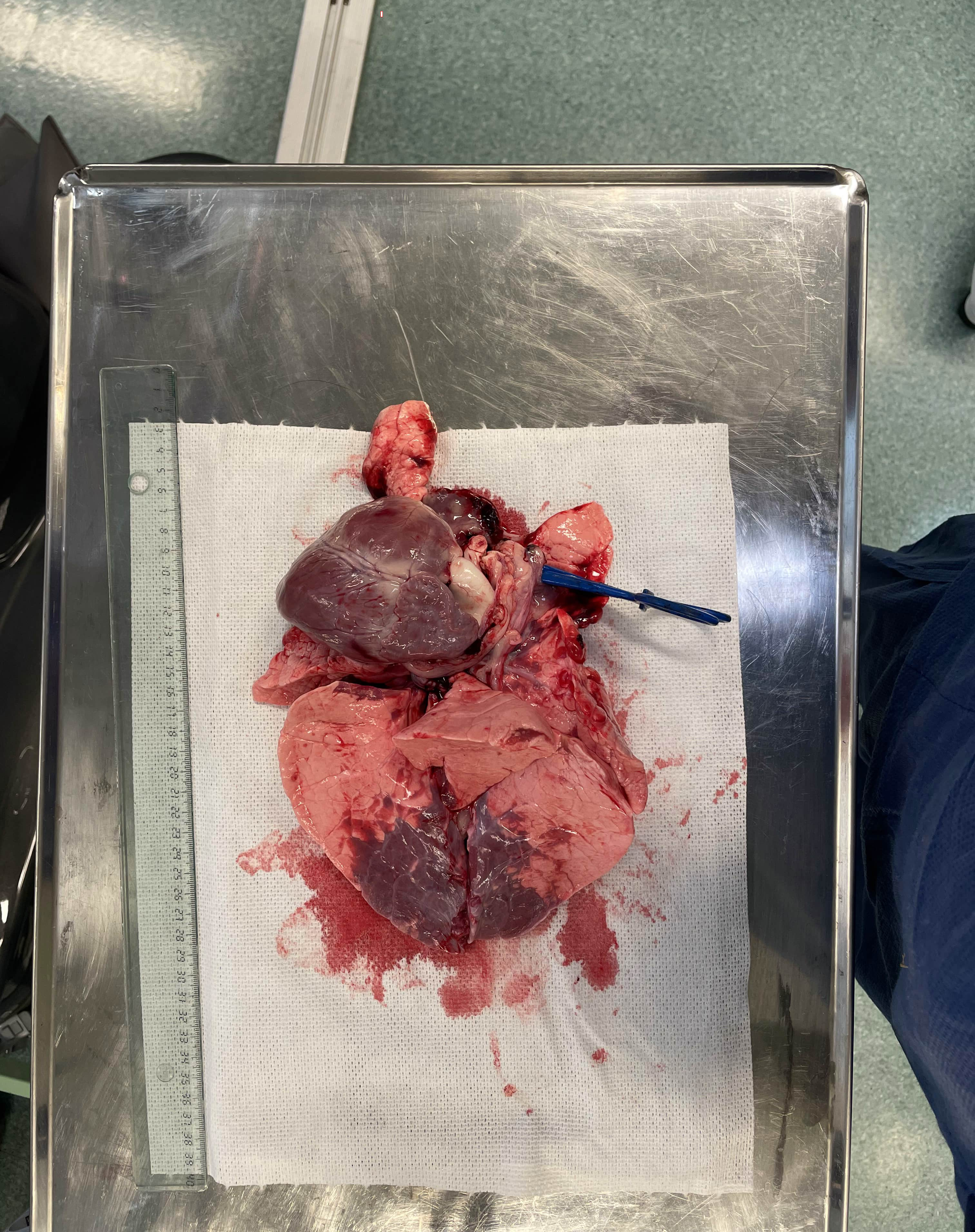

Pig 15
30:2 mode

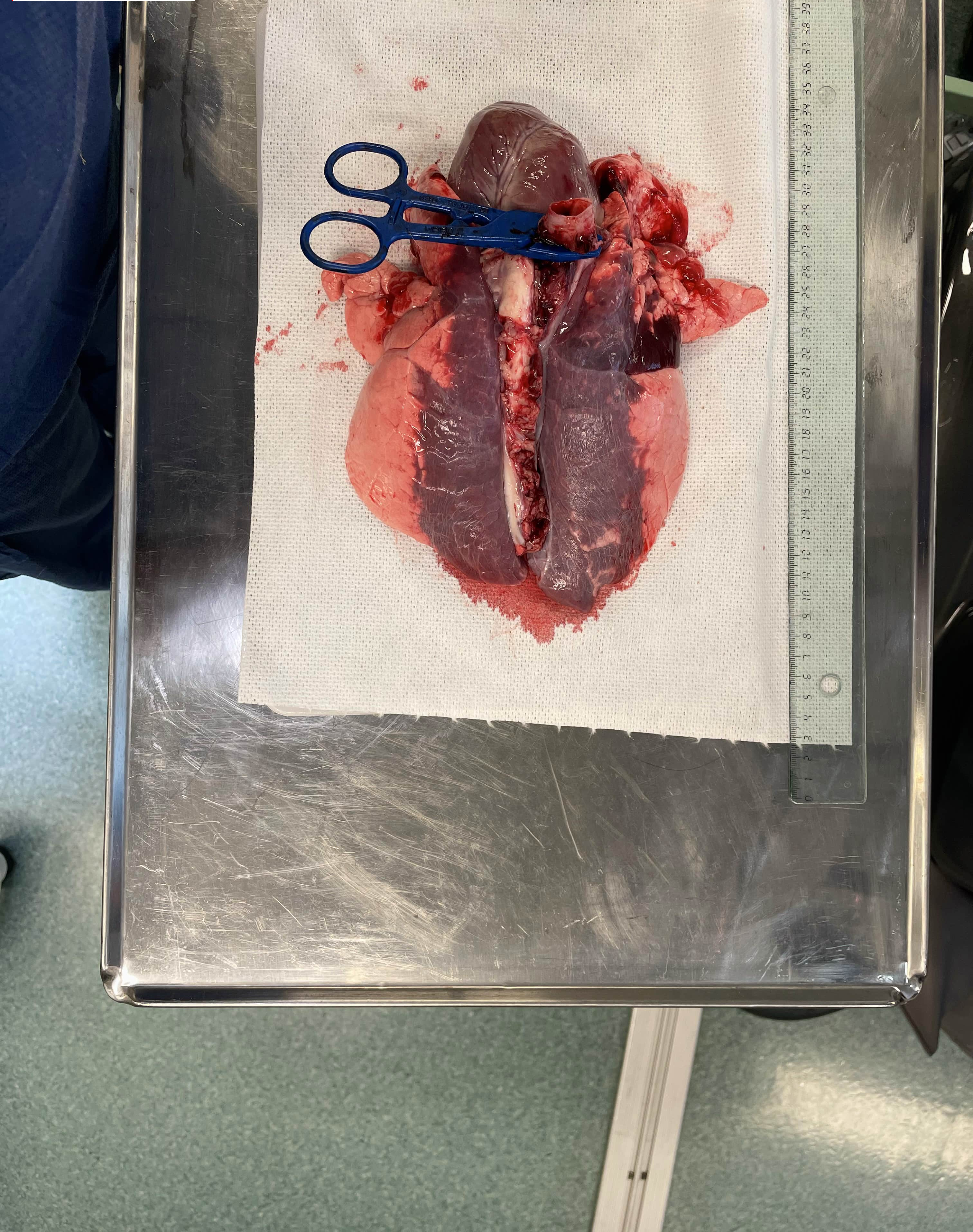

Pig 16
30:2 mode

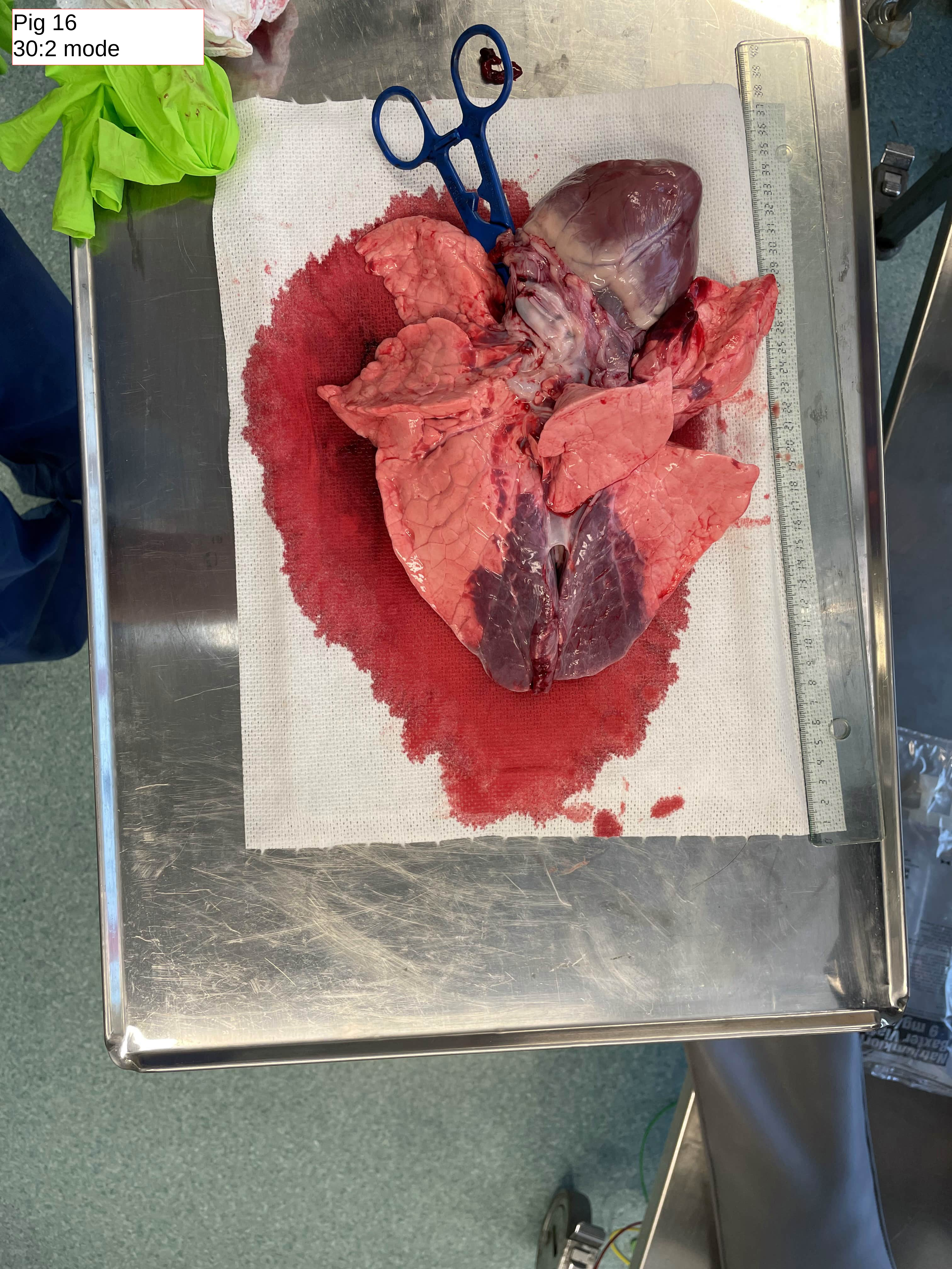

Pig 16
30:2 mode

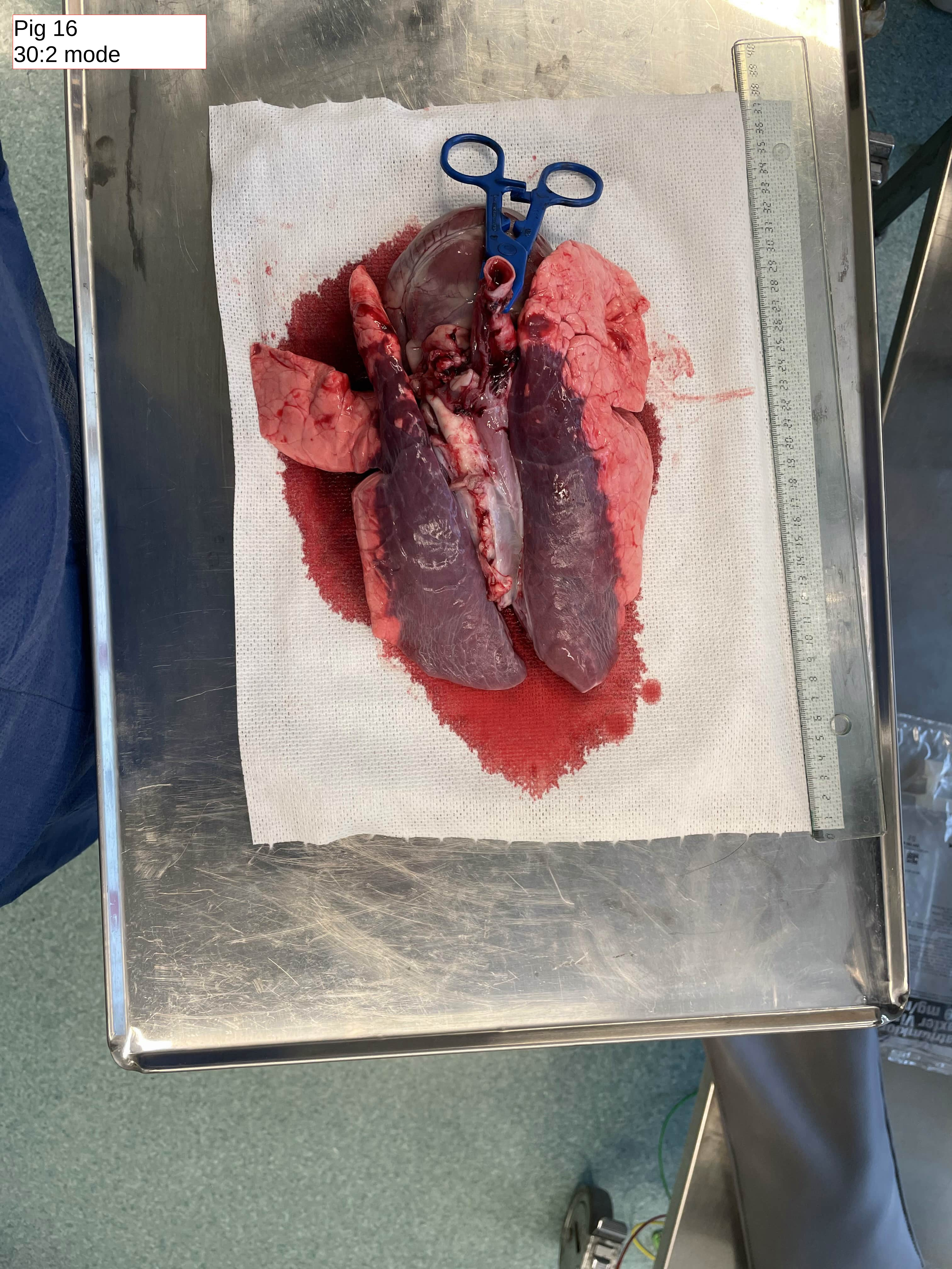

Pig 17
30:2 mode

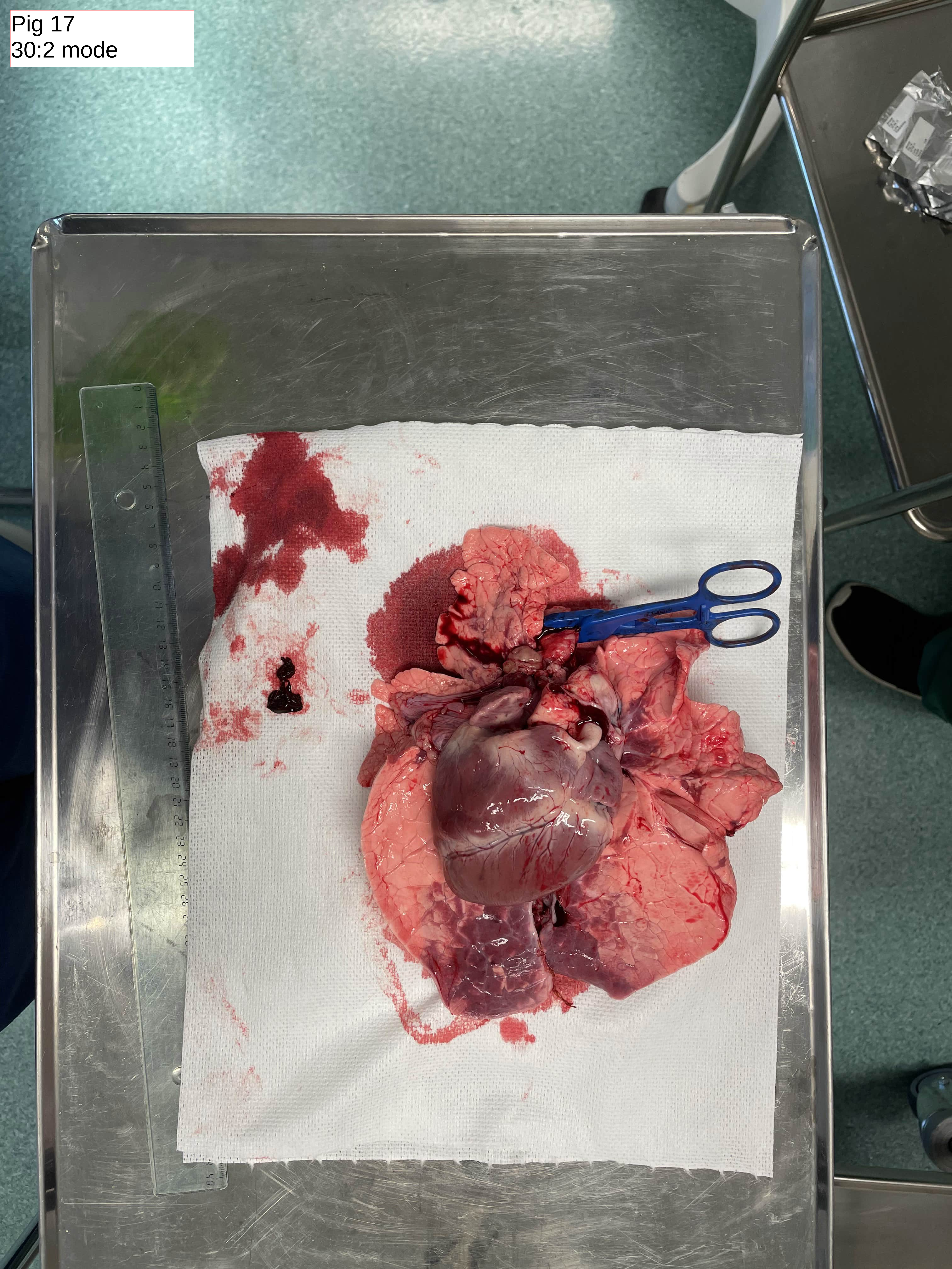

Pig 17
30:2 mode

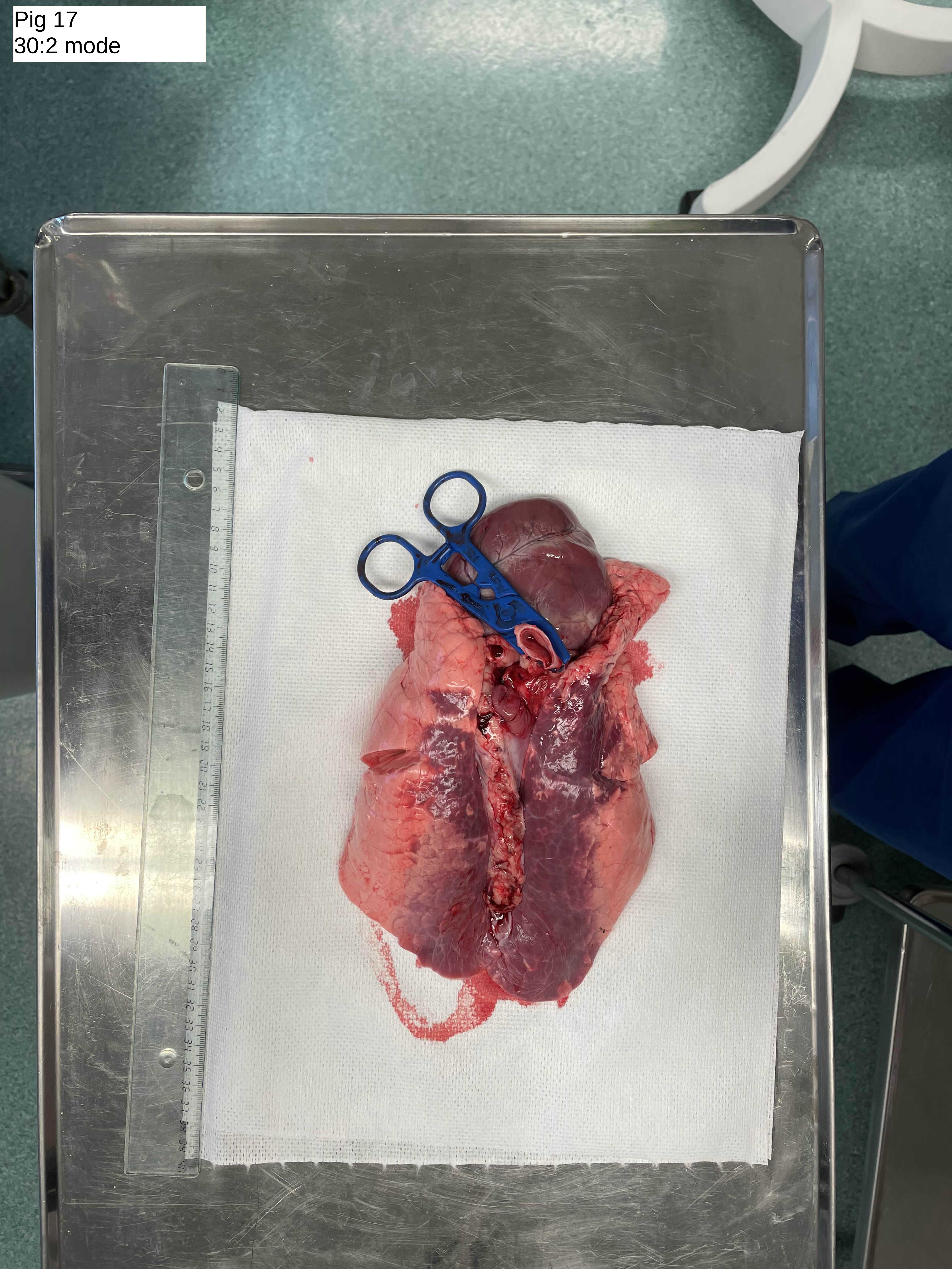

Pig 18
CCC mode

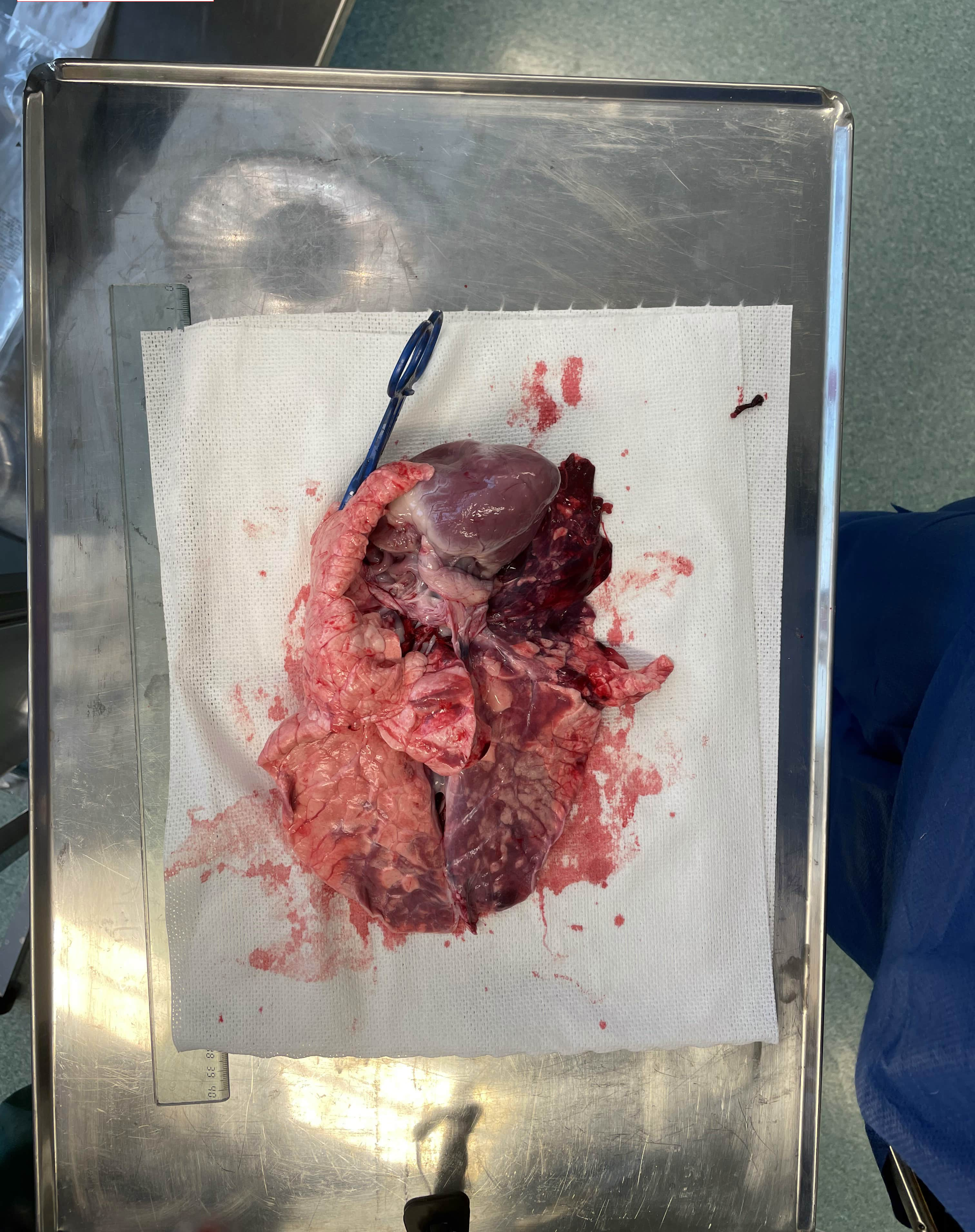

Pig 18
CCC mode

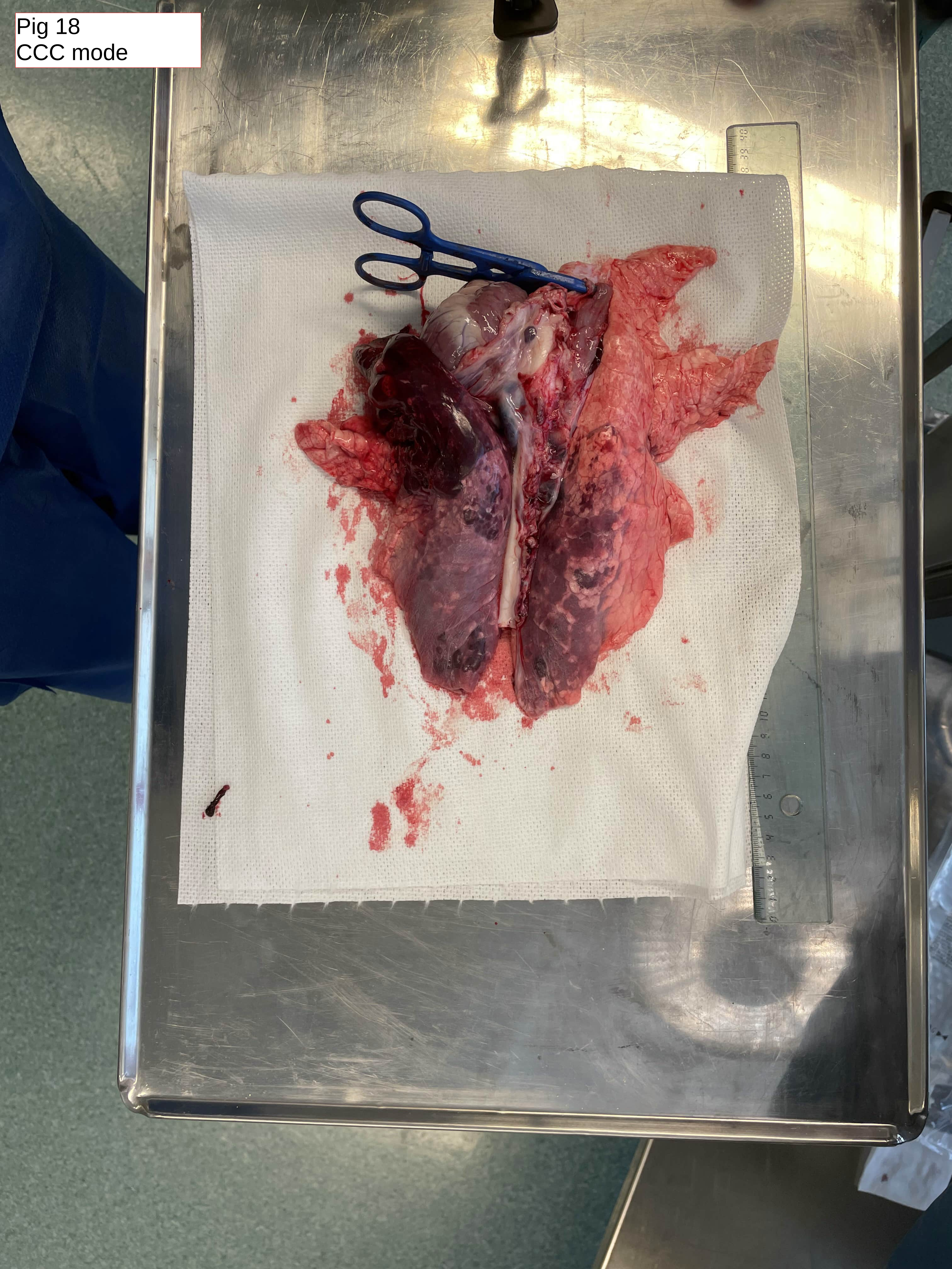

Supplement: Supplementary file 7 — Additional file 7. Lung pictures. [file 40635_2023_559_MOESM7_ESM.pdf]
